# History of Interdental Brushes: Origins, Developments, Perspectives

**DOI:** 10.3290/j.ohpd.c_1800

**Published:** 2025-01-08

**Authors:** Hans Jörg Staehle, Caroline Sekundo

**Affiliations:** a Hans Jörg Staehle Professor emeritus, Department of Conservative Dentistry, Clinic for Oral, Dental and Maxillofacial Diseases, University Hospital Heidelberg, Heidelberg University, Heidelberg, Germany. Conception of the study idea, data analysis and interpretation, drafting of the manuscript.; b Caroline Sekundo Senior Physician, Department of Conservative Dentistry, Clinic for Oral, Dental and Maxillofacial Diseases, University Hospital Heidelberg, Heidelberg University, Heidelberg, Germany. Data analysis and interpretation; critical revision of the article.

**Keywords:** historical milestones of interdental brushes, interdental cleaning, developments in interdental brushes, future indications for interdental brushes

## Abstract

**Purpose:**

To trace the history of interdental brushes (IDBs) from their origins to the present, highlighting their development and future prospects compared to other interdental hygiene aids.

**Methods and Materials:**

A literature search using digital databases, manual reviews and on-site research in museums were carried out.

**Results:**

Although extensive literature exists on toothbrushes, flosses and toothpicks, there has been no comprehensive study of IDBs. Twisted brushes for oral hygiene were mentioned as ear-ly as the late 19th century. The exact origins of their use in interdental spaces remain unclear, but evidence narrows it to the early 20th century. IDBs have been in documented use since at least 1960, with publications emerging in the 1970s. Historically, evaluations of IDBs have been mixed, balancing high expectations with scepticism regarding efficacy and safety. By the early 21st century, IDBs were often considered superior for interdental cleaning. Advances included modifications in brush head designs, handle types, and the establishment of standards like ISO 16409, though these did not always facilitate proper selection and use.

**Conclusion:**

Recent literature still highlights limited evidence-based statements on IDB efficacy, with some questioning their superiority over other aids like dental floss. Consumer-friendly alternatives, such as rubber picks, are sometimes rated higher, however, without allowing for a final assessment. For IDBs to meet the standards of Frugal Dentistry, they must improve oral health, be widely demanded, and cost-effective. Future research should provide more precise indications for IDBs and scientifically sound recommendations for various sizes and designs, ensuring they are easy to use and effective for different interdental spaces.

Oral hygiene aids have been in use for at least 6000 years, with special tools for interdental hygiene, such as toothpicks, documented as early as 3000 BC in Mesopotamia.^26^ Many factors, both systemic and local, can influence the ecological balance in the oral cavity. Oral hygiene remains crucial for preventing carious and periodontal diseases. Preventing gingivitis is the first line of defence against periodontitis. Conventional toothbrushing, which covers the occlusal or incisal, vestibular, and oral surfaces, is a recognised method for preventing gingivitis but is sometimes insufficient.^27,50,51^

In the interdental space, an area particularly prone to plaque accumulation and the development of pathogenic biofilm, specialised tools such as dental floss, rubber picks (IRPs), and interdental brushes (IDBs) are used. IDBs belong to the so-called twisted or spiral brushes. They consist of a twisted wire core covered with side bristles (filaments). They are designed to mechanically influence the ecological niche of the interdental space. Typically, IDBs are passed horizontally across the row of teeth through the interdental space. In special cases, such as patients with fixed orthodontic appliances, they can be guided vertically between the tooth surface and the appliance.

Despite their relative novelty, IDBs have quickly become more popular for interdental cleaning and are well accepted by many patients. Epidemiological studies indicate that approximately 10–20% of people use interdental brushes.^35,43^ However, opinions on their efficacy have been divergent. For instance, in a 1991 study by Kiger et al the authors concluded that IDBs were superior in plaque removal compared to brushing alone or brushing with dental floss. Feedback from study participants indicated that IDBs were preferred due to their simplicity and comfort, suggesting a potential for more consistent and effective interdental cleaning compared to flossing.^42^ This observation was later confirmed by Christou et al (1998), among others.^15^ However, compared to newly developed rubber picks (IRPs), a more recent study by Van der Weijden in 2022 found a preference for IRPs over IDBs.^80^ Heterogeneous statements in the literature on aids for interdental cleaning have existed for decades.

While a search for the history of conventional oral hygiene tools such as toothbrushes, toothpicks, and dental floss reveals a wealth of literature, IDBs stand in stark contrast. This article is the first to comprehensively trace the history of IDBs from their origins to the present day. Based on their development phases, important perspectives for their future use will also be outlined.

## MATERIALS AND METHODS

**Fig 1a and b:**
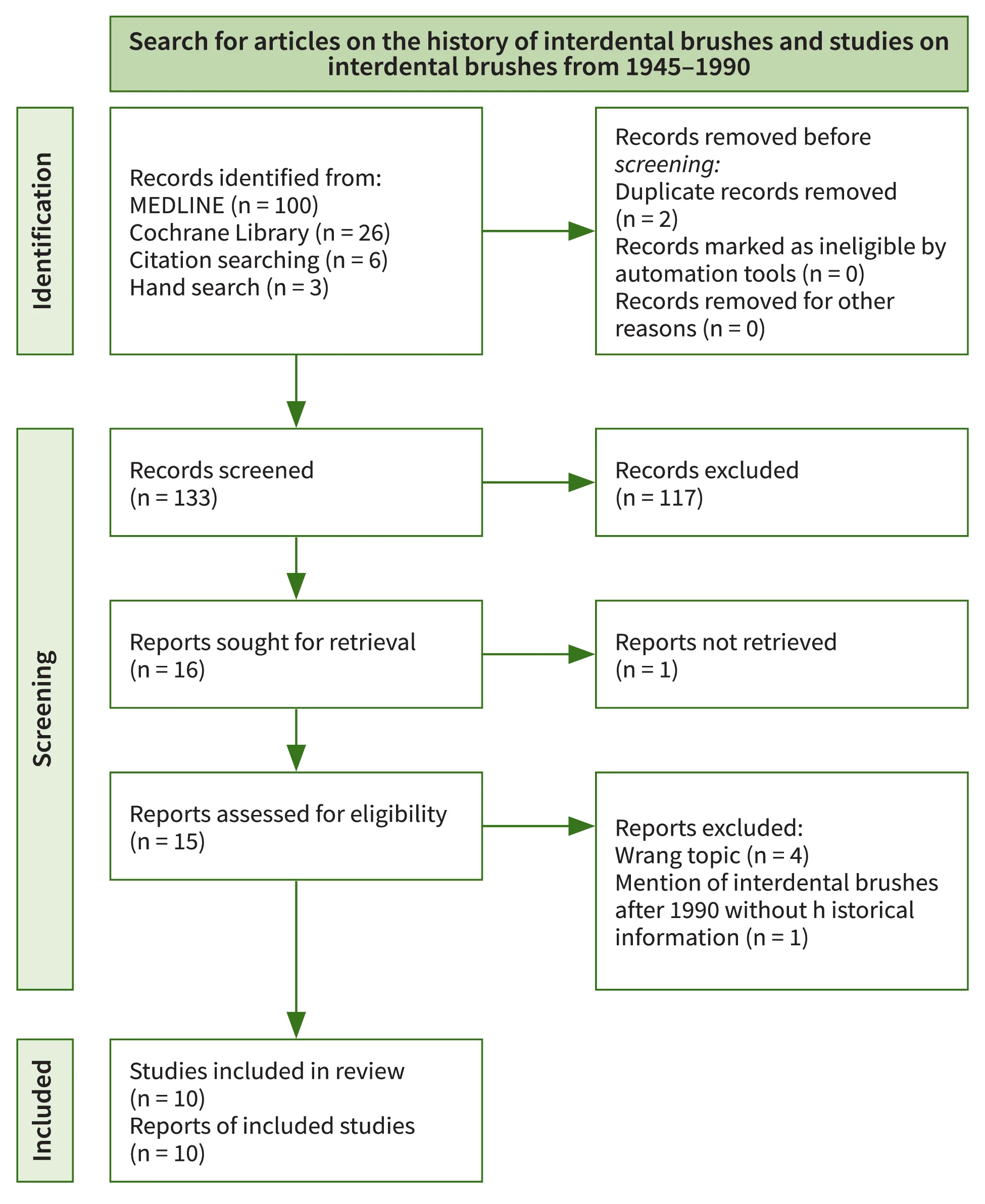

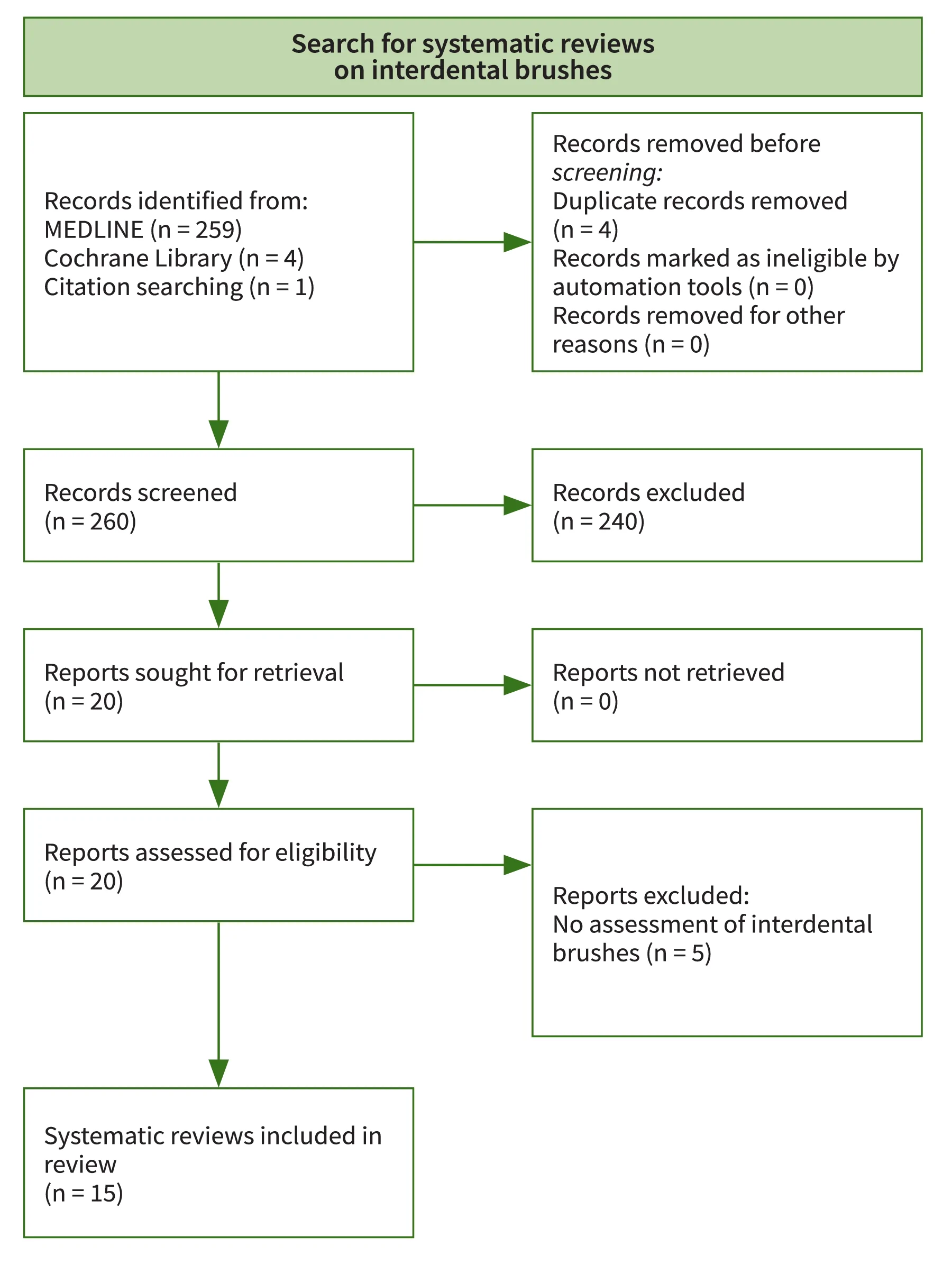
Results of the digital literature search;* (a) *search term for the history of the interdental brushes,* (b)* search term for systematic reviews in interdental hygiene.

The literature search strategies were implemented in MEDLINE via Ovid and in Cochrane Library. The search for historical articles on interdental brushes included the following parameters: Dental Devices, Home Care/hi [History] OR Oral Hygiene/hi [History] OR (histor*.mp. AND [interdental adj2 brush*].mp) OR (interdental adj2 brush*).mp. limited to the years 1945–1990. Inclusion criteria encompassed all article types on the history of interdental brushes (no time limit) and on interdental brushes published from 1945 to 1990. Articles mentioning interdental brushes after 1990 with no historical information were excluded. Additionally, a second search was conducted for systematic reviews using the parameters: (Dental Devices, Home Care/ OR Oral Hygiene/ OR [interdental adj2 brush*].mp.) limited to ‘systematic review’ OR (Dental Devices, Home Care/ OR Oral Hygiene/ OR [interdental adj2 brush*].mp.) AND systematic review.mp (see Figs 1a and 1b). Only systematic reviews assessing clinical outcomes of interdental brush use were included, all other article types were excluded.

Further historical research included hand searches in books and journals, as well as on-site research in museums (Dental Museum Zschadraß, Germany; Brush Museum Bechhofen, Germany; Brush Museum Todtnau, Germany). This involved examining exhibits, product instruction leaflets, brochures, company brochures, historical company advertisements, and conducting interviews with contemporary witnesses.

## RESULTS

### The Beginnings of IDBs (Up To and Including 1990)

#### The situation before 1970

**Fig 2:**
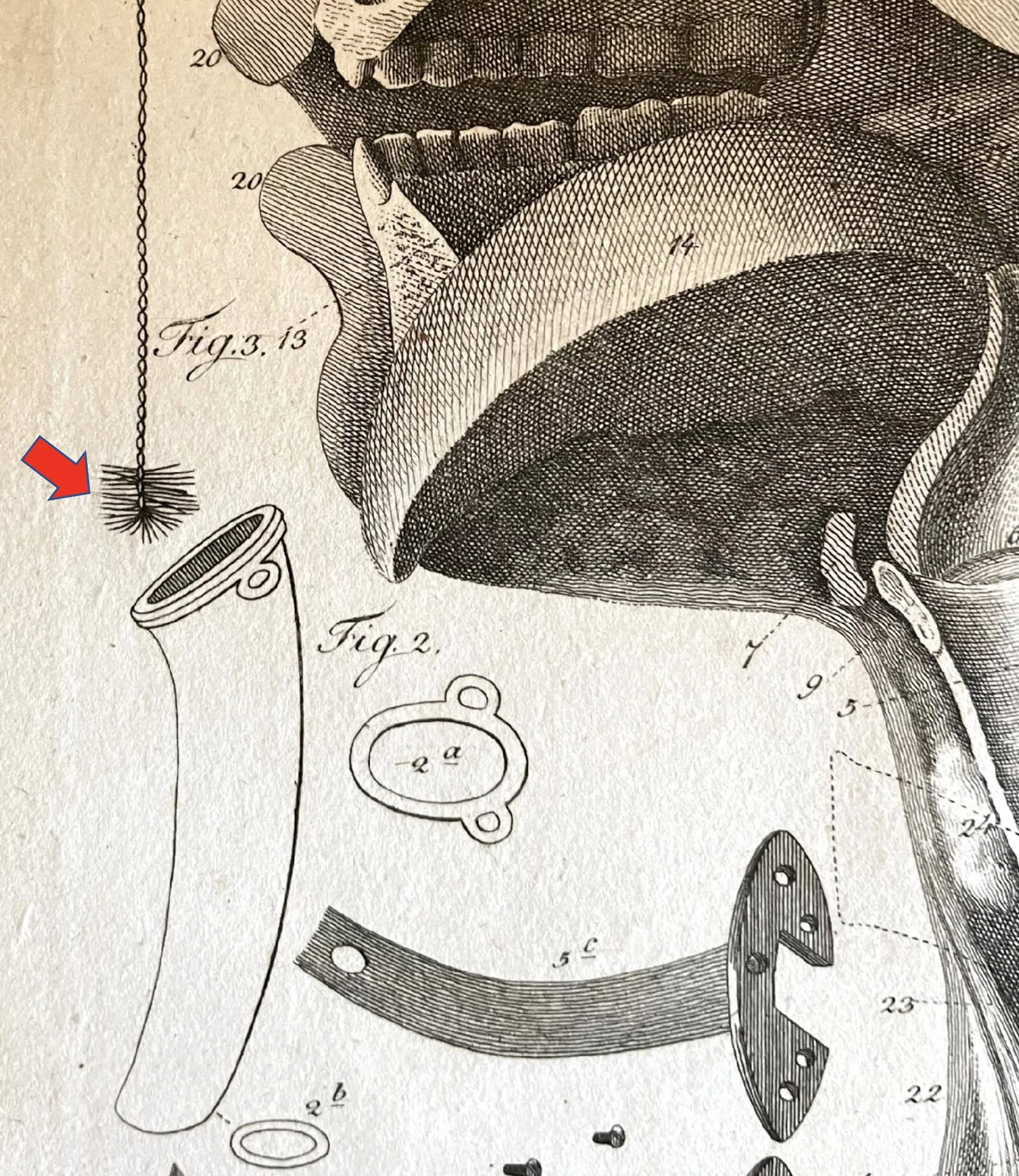
Cleaning an intubation tube (Brétonnean’s silver tube) with a small, twisted brush (see arrow) during a tracheotomy treatment. In: Gr. Herzogl. Sächs. Priv. Landes-Industrie-Comptoirs: chirurgische Kupfertafeln, 38. Heft, Tafel CLXXXVII-CXLL, Weimar 1827, Germany. Source: Andreas Haesler, Dental Museum Zschadraß, Germany.

The origins of IDBs (twisted brushes/spiral brushes) are somewhat obscure. Such brushes, where the bristles are held in twisted wires, were developed and offered by the beginning of the 19th century at the latest, and possibly as early as the second half of the 18th century during industrialisation. They were mentioned in Krünitz’s Encyklopädie in 1810 and described in detail in 1851 in a book published in Zurich on craftsmen’s and artists’ workshops, particularly regarding their manufacture and use (eg, for cleaning pipe tubes and bottles) (cited in Bock, 1983^9^). These brushes were used for medical purposes early on. For example, a small, twisted brush for tracheotomy treatment (cleaning an intubation tube) is depicted in a German collection of pictures for practical surgeons from 1827 (Fig 2).^31^

Twisted brushes were also used to clean a chemical analysis and extraction apparatus developed by the chemist Franz von Soxhlet (1848–1926) in 1879, which consisted of various straight and angled tube systems of different diameters (Soxhlet, 1879^72^). They came into circulation under the name Soxhlet brushes (Bock 1983^9^). Brushes of this type were varied in shape for a variety of purposes. In addition to bottle brushes, cylindrical brushes (angular, cylindrical shape at the front), test tube brushes (rounded, cylindrical shape at the front) and spout brushes (conical shape for cleaning tubular or funnel-shaped attachments of vessels) were available.

**Fig 3:**
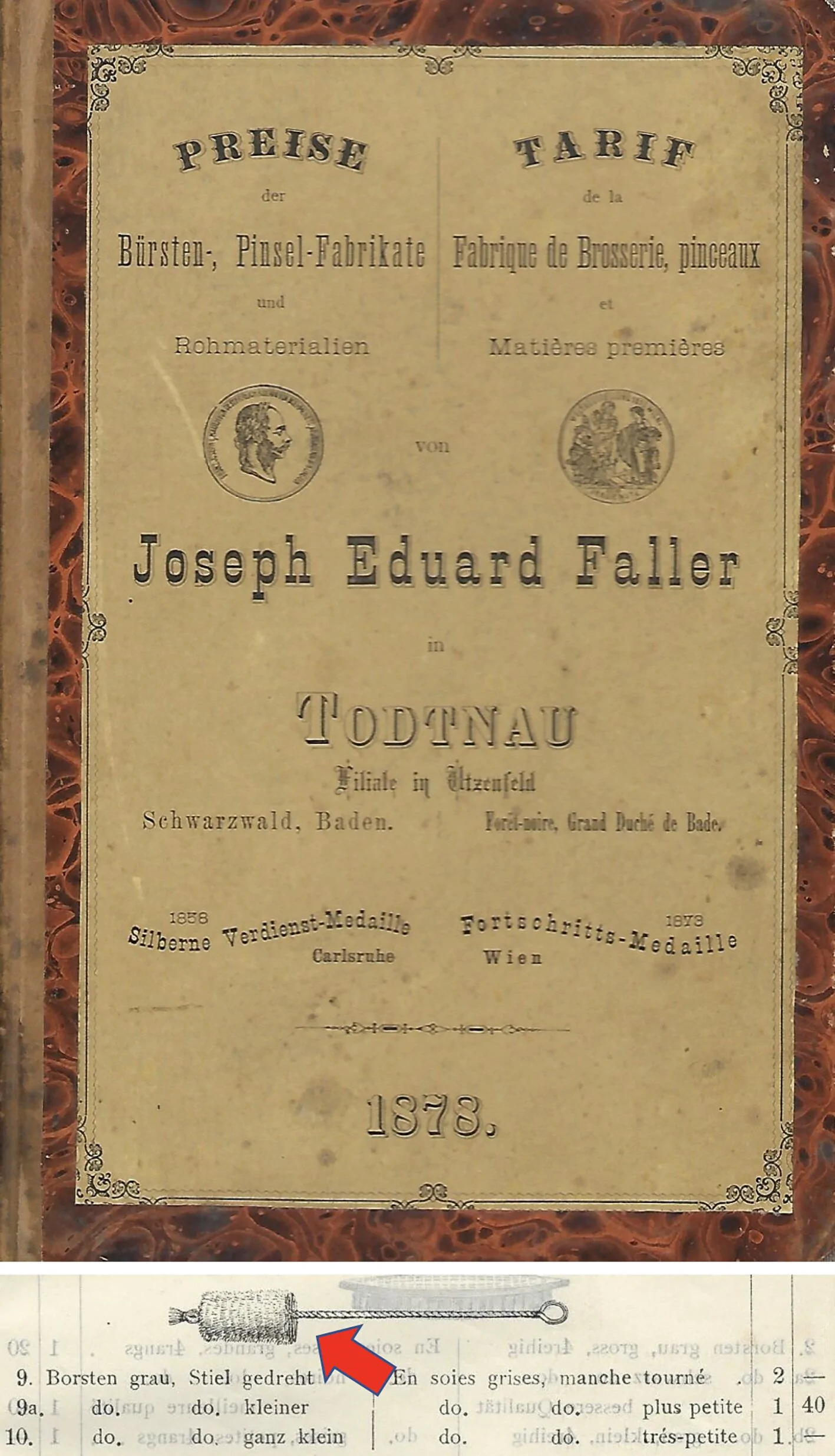
Twisted brushes in different sizes (normal, small, very small). In: Joseph Eduard Faller, Preisliste für Bürsten, Pinsel-Fabrikate, Todtnau 1878. Source: Benno Dörflinger, Brush Museum Todtnau, Germany.

Twisted brushes could be made in various sizes. In an illustrated price list published by the company Joseph Eduard Faller (Todtnau, Germany) in 1878, specific reference was made to different sizes, with ‘small’ and ‘very small’ models being mentioned alongside normal designs (Fig 3).^25^ This also broadened the range of applications (eg, for cleaning the inner surfaces of small bottles, sleeves, nozzles and hoses).

**Fig 4:**
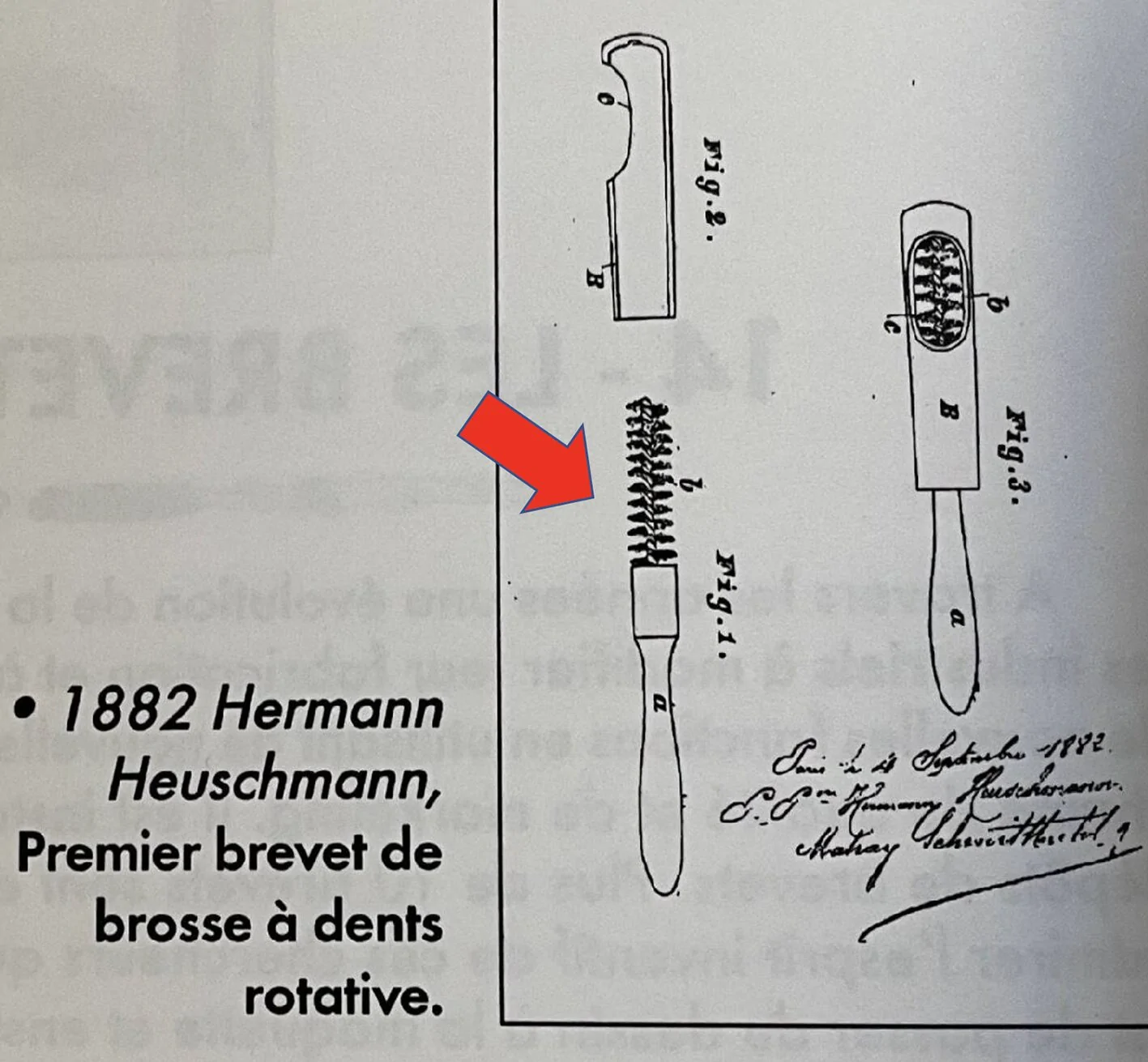
Toothbrush in the form of a twisted brush that can rotate within a partially open outer shell. First described in 1882 by Hermann Heuschmann. In: Bogopolsky S. Itineraise Culturel et technologique de la brosse à dents. Èditions des écrivains, 1999, p. 114; Bogopolsky S. La brosse à dents. Èditions CdP, Paris 1995, 1999, p. 89. Source: Andreas Haesler, Dental Museum Zschadraß, Germany.

**Fig 5:**
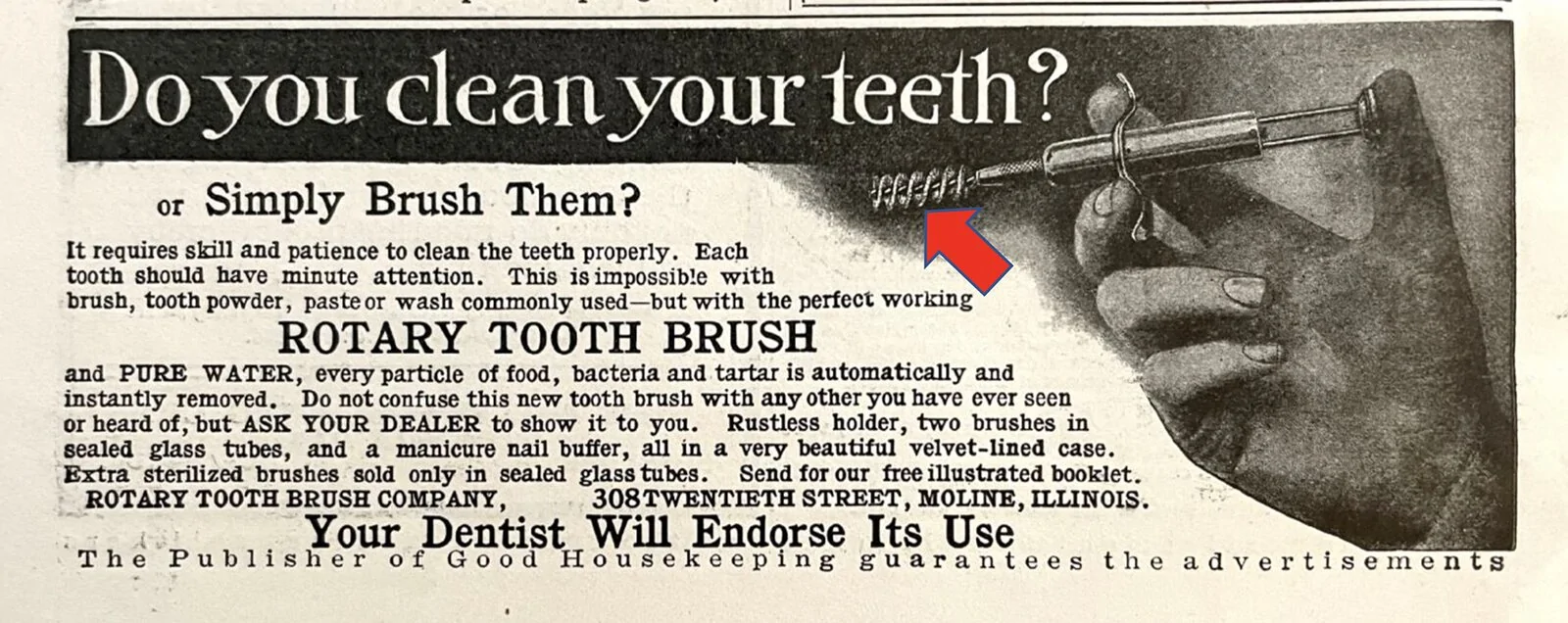
Twisted toothbrush in a water irrigation apparatus from 1903. In: Rotary Tooth Brush Company, Moline, IL, USA (advertisement). Source: Good Housekeeping – The Housekeepers’ Directory, Canada, 12 July 1903. Source: Andreas Haesler, Dental Museum Zschadraß, Germany.

In 1882, Hermann Heuschmann described twisted brushes with a cylindrical shape in longitudinal section for the purpose of cleaning entire rows of teeth. Heuschmann mounted them rotatably in partially open protective hoods (Fig 4).^10,11^ Such devices, which can be considered early precursors of mechanical toothbrushes,^23^ had various modifications, such as the ‘Rotary Tooth Brush’ combined with water rinsing, offered in a 1903 advertisement by the Rotary Tooth Brush Company (Moline, IL, USA) (Fig 5).^61^

**Fig 6:**
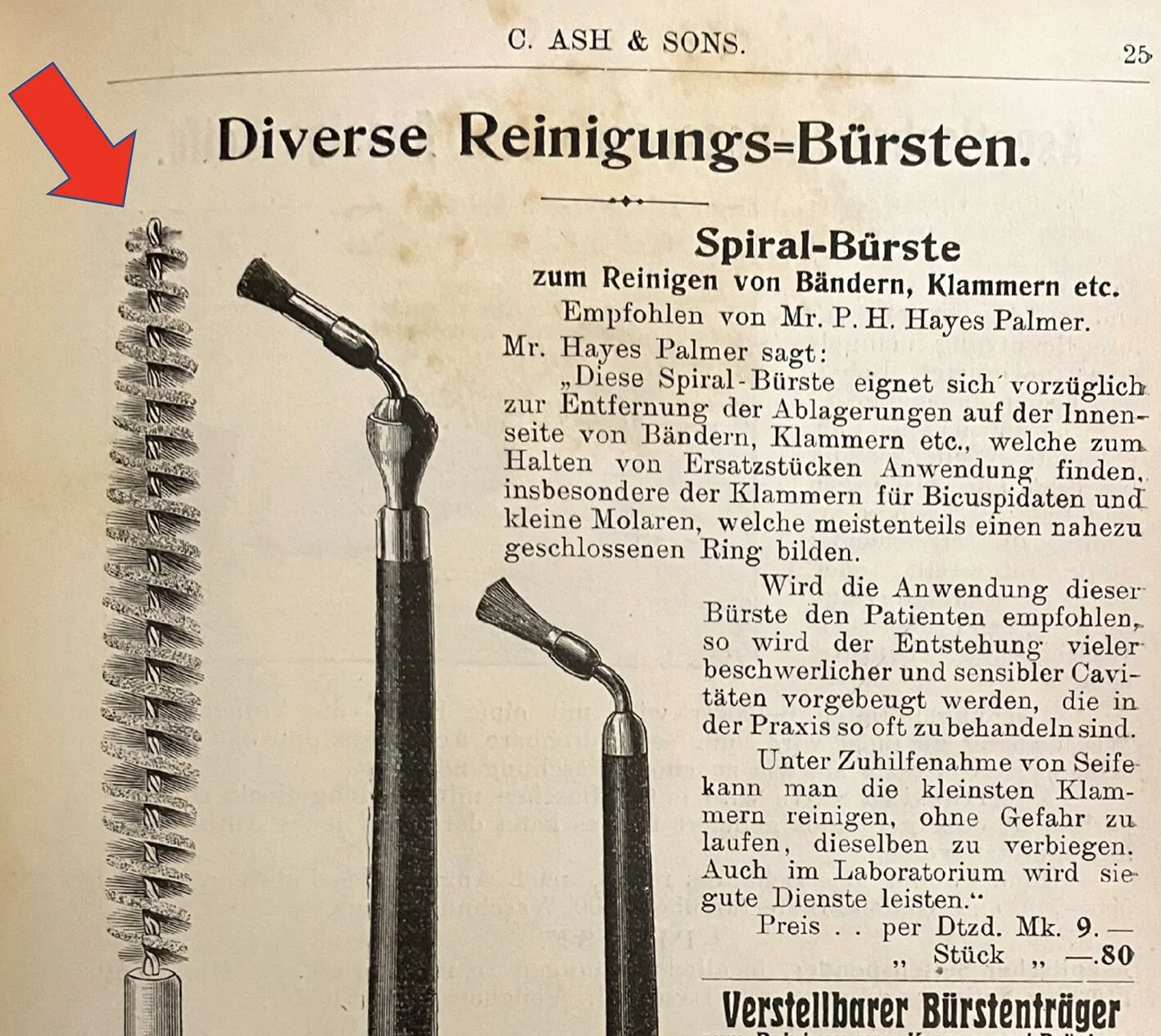
Various non-rotating cleaning brushes (left: twisted brush, middle and right: single-tuft brushes) with variable applications, such as cleaning the inside of bands and ring-shaped prosthesis clasps. In: C. Ash & Sons: Zahnpasten, Zahnpulver, Mundwasser, Zahnbürsten. Katalog-Abteilung VIII, 5th edition, 1909. Source: Andreas Haesler, Dental Museum Zschadraß, Germany.

**Fig 7:**
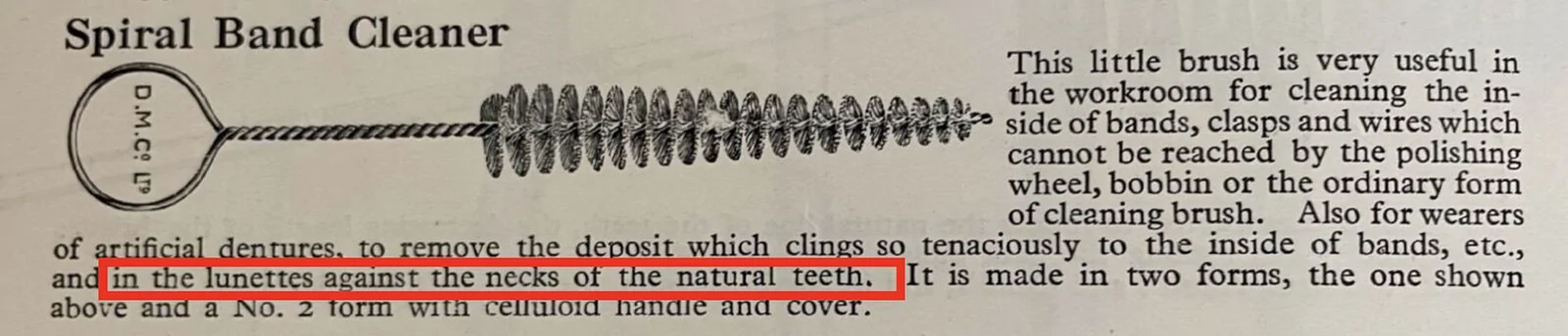
Twisted toothbrush (spiral brush), which not only cleans the inside of bands and prosthesis clasps but also removes deposits in the semicircular or arch-shaped areas (‘lunettes’) between artificial teeth of a partial denture and the necks of natural teeth. This is only possible if used not lengthwise along the dental arch but across (interdentally). Additionally, it must be delicate enough to pass through narrower areas. In: The Dental Manufacturing Company Ltd. (DMC) London, 1927, p. 66. Source: Andreas Haesler, Dental Museum Zschadraß, Germany.

At the beginning of the 20th century, the desire for targeted cleaning of hard-to-reach areas of teeth, as well as elements of partial dentures (eg, clasps), was also reflected in the range of delicate single-tufted brushes and twisted brushes. An example of this can be seen in a product catalogue from C. Ash & Sons (London, England) from 1909.^4^ A small spiral brush with a convex longitudinal section was also presented, which was primarily intended for cleaning ring-shaped denture clasps on premolars and molars, but which was also suitable for passing larger interdental spaces due to its dimensions (Fig 6). This idea was taken up in 1927 in a brochure from Dental Manufacturing (DMC, London, England) which presented a twisted brush with a conical design in longitudinal section, suitable for cleaning deposits in the arched areas (‘lunettes’) between artificial teeth of a partial denture and the necks of natural teeth (Fig 7).^19^ Such brushes, positioned transversely to the row of teeth, inspired the later use of interdental brushes.

**Fig 8:**
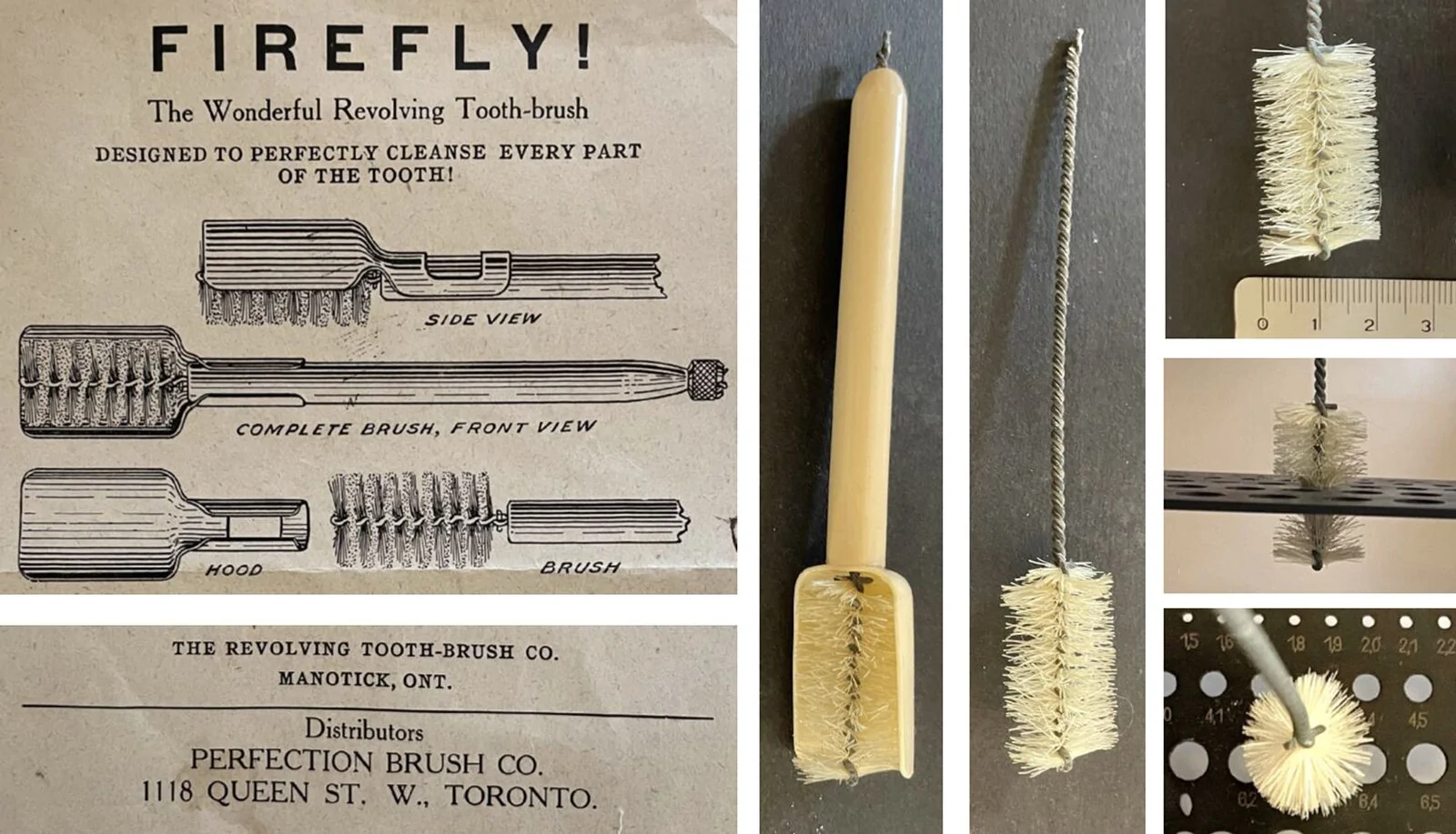
Firefly Tooth Brush (twisted brush:) (a) perfection on Brush Toronto (company brochure). Firefly – the Wonderful Revolving Toothbrush (date range 1930 to 1940 according to Research Collection Catalogue of the Museum of Health Care at Kingston. https://mhc.andornot.com/en/permalink/artifact13766. Accessed on 10 December 2023). Distributors: Perfection Brush Co., Toronto. (b) Brush with hood (usable only lengthwise along the dental arch); (c) brush without hood (also usable across the dental arch); (d) brush dimensions: outer diameter 20 mm, wire core diameter 2.5 mm. (e) and (f) PHD value: 6.3 mm. The exhibits shown in (b) to (f) are located in the Dental Museum, Zschadraß, Germany; Photos: Hans Jörg Staehle.

**Fig 9:**
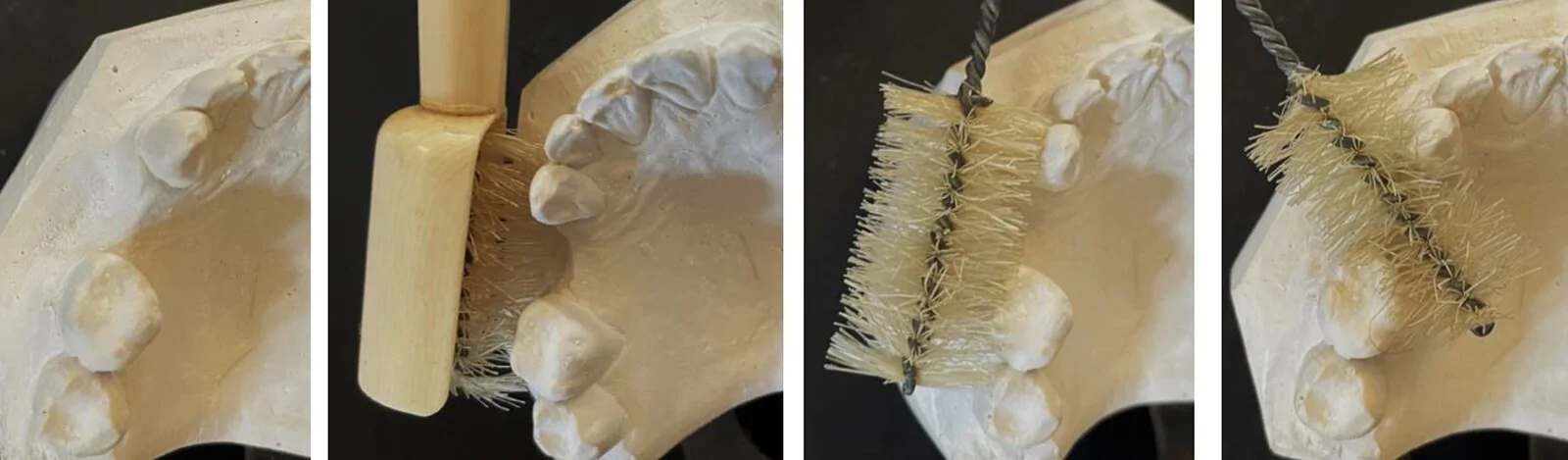
Demonstration of the use of a Firefly Tooth Brush: *(a) *untreated dentition with gaps; *(b) *position of the spiral brush (with hood) lengthwise along the dental arch; *(c)* Position of the spiral brush (without hood) lengthwise along the dental arch without capturing proximal surfaces; *(d)* Position of the spiral brush (without hood) across the dental arch capturing proximal surfaces. The exhibits shown are located in the Dental Museum, Zschadraß, Germany; Photos: Hans Jörg Staehle.

**Fig 10:**
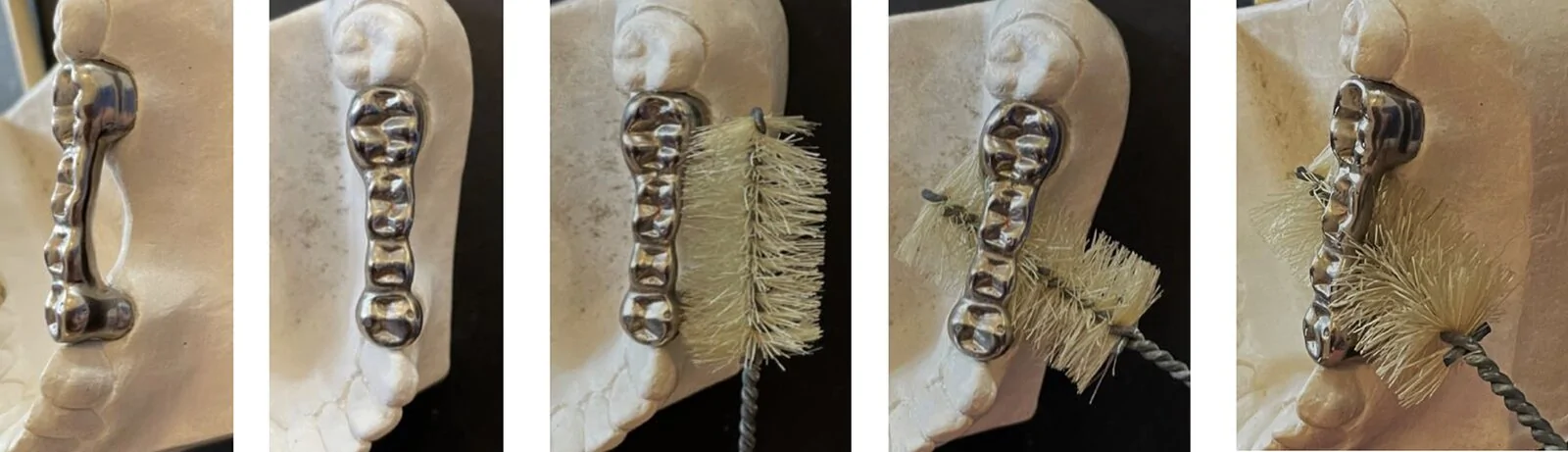
Demonstration of the use of a Firefly Tooth Brush. *(a)* dentition with a sanitary bridge, lateral view; *(b)* occlusal view; *(c) *position of the spiral brush (without hood) lengthwise along the dental arch without capturing proximal surfaces;* (d) *position of the spiral brush (without hood) across the dental arch capturing proximal surfaces; occlusal view; *(e) *position of the spiral brush (without hood) across the dental arch capturing proximal surfaces; lateral view. The exhibits shown are located in the Dental Museum, Zschadraß, Germany; Photos: Hans Jörg Staehle.

In the 1930s to 1940s, twisted brushes were marketed under the company name ‘Firefly’ (Perfection Brush, Toronto ).^52^ The product ‘Pro-Bel’ (La Cie Anglo-French Drug, Montreal), patented in 1941, followed a similar concept,^47^ as well as the product ‘Rolli’, introduced in 1956.^65^ The Firefly toothbrush (Fig 8a) could be handled with and without a cap (Figs 8b and 8c). It had an outer diameter of 20 mm and a wire core diameter of 2.5 mm (Fig 8d). Its passage hole diameter (PHD) value (see ‘Standards for IDBs’) was 6.3 mm (Figs 8e and 8f). The brush was probably used with or without a cap, mainly in the longitudinal direction of the rows of teeth (Figs 9a–9c). In the case of teeth with gaps or under the sanitary bridges known since the end of the 19th century,^46^ it was also possible to position the brush transversely (Fig 9d and Figs 10d and 10e).

The exact date and inventor of the first twisted brushes used in interdental spaces are unknown. Delicate shapes of various products intended for other purposes may have been used for interdental cleaning. Against the background of the available results, it is only possible to narrow down the time period to the first half of the 20th century.

**Fig 11:**
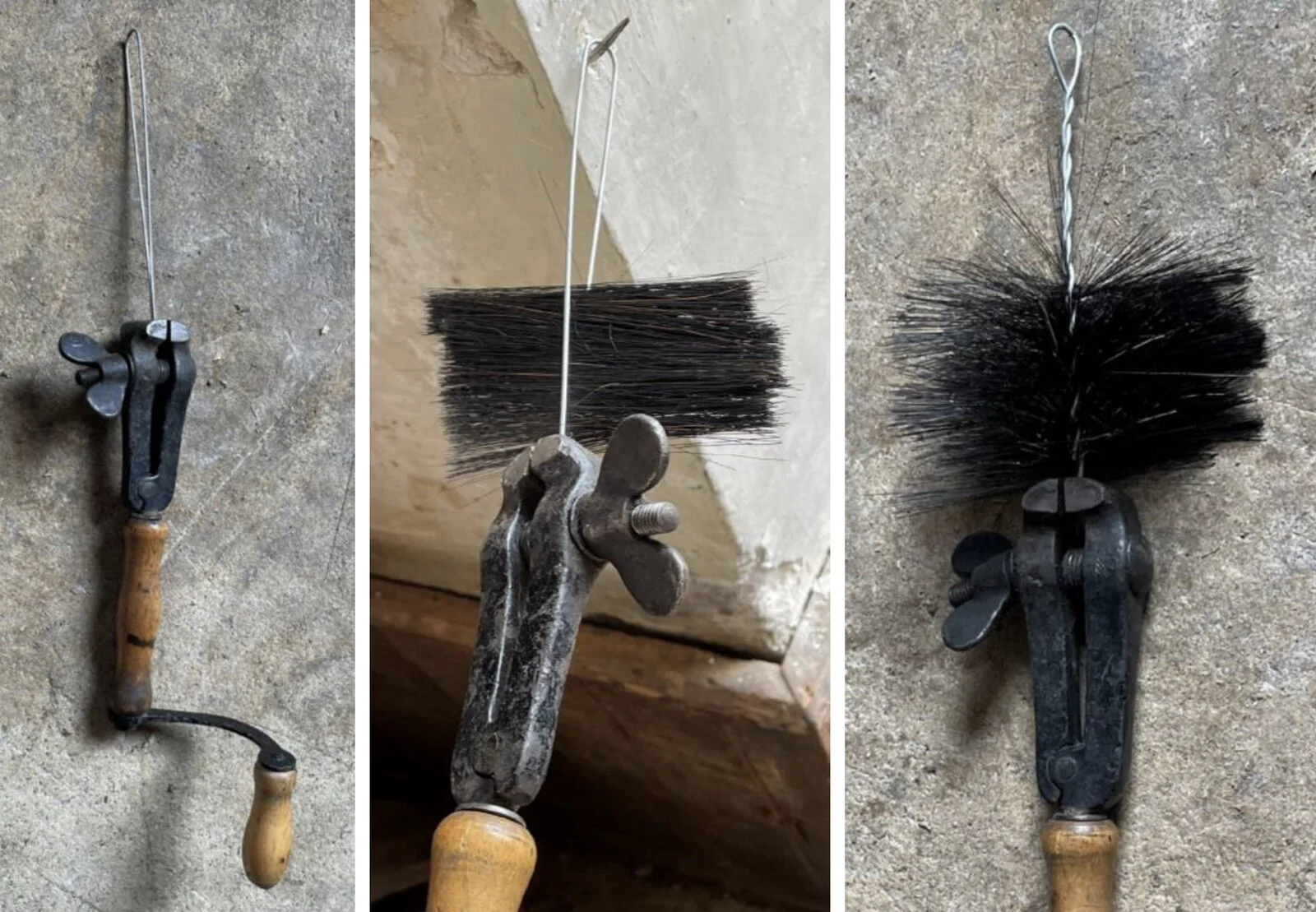
*(a)–(c) *Hand crank for the manual production of twisted brushes, around 1900. Brush Museum (Todtnau, Germany). Photos: Hans Jörg Staehle.

**Fig 12:**
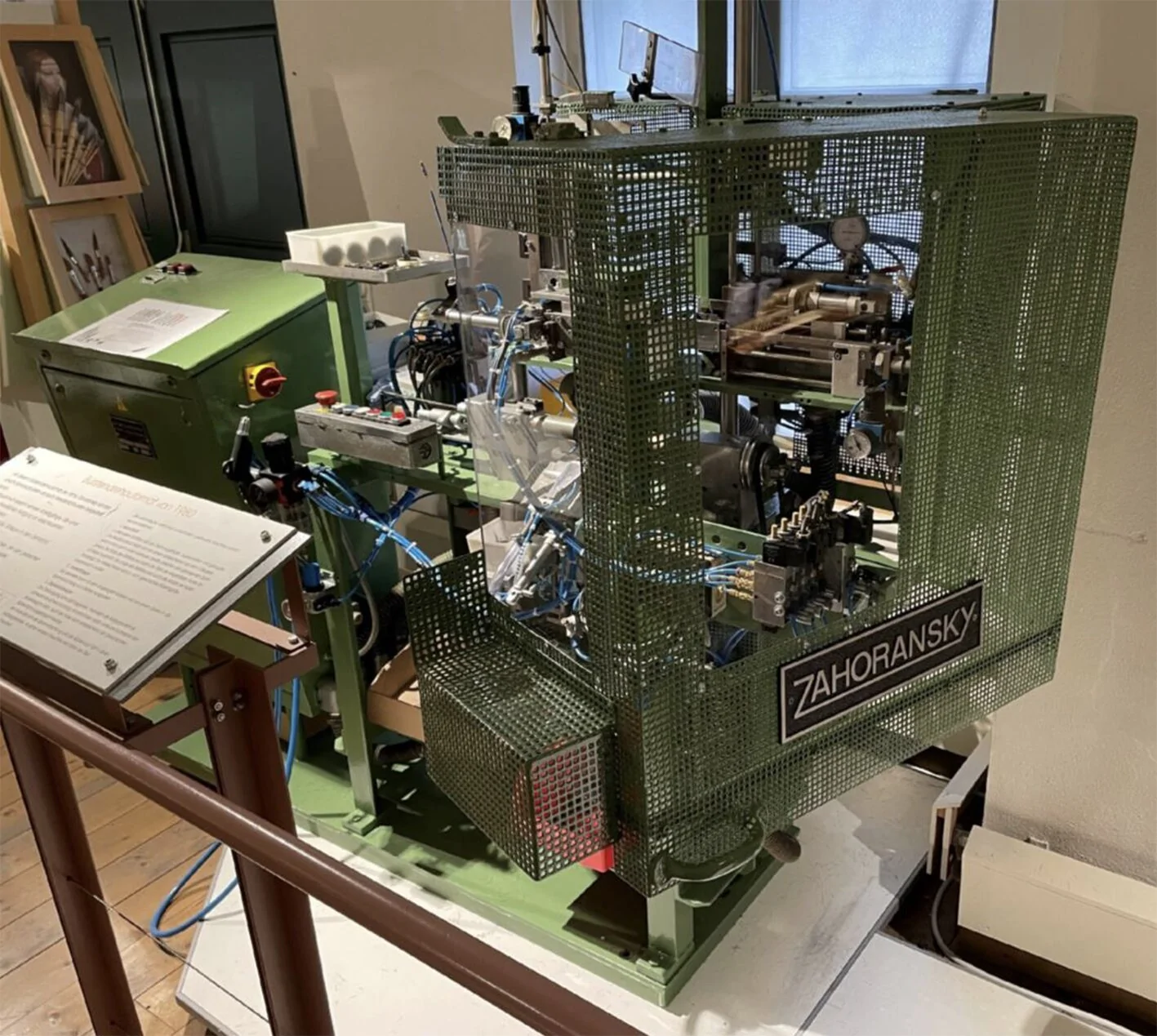
Zahoransky brush twisting machine from 1980. Brush Museum Bechhofen, Germany. Photo: HJ Staehle.

Initially, twisted brushes were handmade using rotating hand cranks (see Fig 11). In the first half of the 20th century, mechanical lathes with shearing devices for the filaments allowed for variable brush contour designs. Filaments were initially natural bristles, later replaced by nylon. In 1975, German mechanical engineer Ulrich Zahoransky88 developed a two-part machine (type KD/A – KVL) specifically for producing cylindrical interdental brushes (6 mm diameter × 30 mm length). Figure 12 shows a brush turning machine from 1980.89

#### Situation from 1970 to 1980

The sections ‘Situation from 1970 to 1980’ and ‘Situation from 1980 to 1990’ present the results of the literature search for articles that can provide information on the history of IDBs. On the basis of the first search term, 10 results were obtained that are exclusively attributable to the last part of the search term (see Fig 1a).

In 1970, the first scientific study involving IDBs was published by Gjermo and Flötra (Oslo, Norway).^29^ The authors differentiated between dental floss (no manufacturer information), toothpicks (toothpicks from the Norwegian company Jordan) and single-tufted brushes (Tandex Solo single-tufted brush, without manufacturer information). They also presented a picture of a spiral brush labelled ‘interdental brush’ (also from the Norwegian company Jordan). This brush was cylindrical in longitudinal section. It resembled a small bottle or tube cleaner and merged seamlessly into the central wire core, which served as a handle. The inscription ‘Jordan interdental brush no. 3’ indicates that there were different sizes.

The authors noted that Kvam (1966) and Waerhaug (1967) had previously mentioned various aids for interdental cleaning, including ‘interdental brushes’. In the study by Gjermo and Flötra, IDBs were used for 4 weeks by six people with ‘wide open’ interdental spaces due to periodontal destruction. The brushes proved to be more effective at plaque removal than dental sticks and dental floss. However, detailed information on the test subjects’ medical history, the recorded sizes of the interdental spaces and the selection and application criteria for the IDBs was largely lacking.

In 1976, Wolffe (London, England) published a study in which dental sticks (Inter-Dens from Navec International), single-tuft brushes (Interspace from Daily Use Ltd.) and waxed dental floss (from Johnson & Johnson Ltd.) were used.^86^ No significant differences were found between these devices in terms of plaque removal. Wolffe referred to the study by Gjermo and Flötra, among others, and falsely claimed that they used an ‘interspace brush’ with a tuft. However, Gjermo and Flötra explicitly described a real ‘interdental brush’. Wolffe’s work later appeared in digital searches under the misleading keyword ‘interdental brush’, probably due to the similar terms (interspace brush vs. interdental brush) for different products.

Waerhaug (Oslo, Norway) presented another study in 1976, which is still one of the most fundamental works on IDBs today.^82^ He illustrated two cylindrical IDBs in longitudinal section for interdental spaces of different sizes, which were marketed by the Norwegian company Jordan. The wire core of the smaller brush had a thickness of about 1 mm, with side bristles of 1.5 mm each, resulting in a total diameter of about 4 mm. The wire core of the larger brush was also about 1 mm, with side bristles of 3 mm each, resulting in a total diameter of about 7 mm.

The author analysed teeth scheduled for extraction from two groups of patients: those who had never used IDBs (31 extracted teeth) and those who had used IDBs for up to 14 years (36 extracted teeth). He examined radiographs of 24 patients who had been using IDBs for a decade or more. Before extraction, all teeth were cleaned with interdental brushes only and the position of the gingival margin was marked.

Using the extracted teeth, he was able to show that the IDBs removed plaque from the coronal tooth surfaces (vestibular and oral) and the subgingival areas, depending on the length of the lateral bristles or filaments. Waerhaug had observed that the wire core of IDBs was always at the level of the gingival margin, regardless of the condition of the respective periodontium (inflammation, pockets, etc.). He wrote: ‘Since the bristles of the large interdental brush are about 3 mm long, they can at the maximum penetrate to the same depth below the gingival margin and remove plaque as was demonstrated in Fig 3-6. The depth of insertion of the small interdental brush is about 1½–2 mm (Fig 2)’. Taking a summary view of all the patients examined, he assumed a subgingival plaque removal of about 2 to 2½ mm. In the case of an irregular tooth or root surface, plaque removal by IDBs was reduced. He explained this as follows: ‘By studying Fig 7 it becomes clear that within the pocket the bristles must work parallel (sic!) with the tooth surface; this explains why they cannot remove plaque in narrow pits’. He explained the advantage of IDBs compared to dental sticks as follows: ‘The interdental brush has an effect below the gingival margin to the same extent as it also has on the buccal and lingual surfaces. This is why it is superior to the toothpick’. He summarised his comparative radiographic studies as follows: ‘A comparison of the bone height in the radiographs taken before, and 10 years or more after the introduction of the interdental brush revealed no loss of attachment which could relate to the use of the brush. The loss of attachment which was observed in the cases shown in Fig 5 and 6, obviously was not caused by the brush but by the plaque it had not been able to remove.’

Waerhaug referred to 14 years of clinical experience with IDBs and also showed an illustration. His manuscript was accepted on 1 May 1975. Since the data had to be collected and analysed, the manuscript written, submitted and subjected to the peer review process, it can be assumed that the paper was written in 1974. This in turn means that Waerhaug had been using IDBs clinically on patients since 1960 at the latest. This assessment corresponds with the findings of Van der Weijden et al who pointed out in 2008 that IDBs were commercially available from the 1960s onwards.^79^ It is also consistent with a paper by Waerhaug from 1959, in which he discussed interdental care for periodontitis prophylaxis and presented various aids, but did not yet mention IDBs.^81^

In 1977, Nayak and Wade (London, England) presented a new IDB called ‘Proxabrush’ from the American company Butler, which differed from the previously known products.^54^ The brush was not cylindrical in longitudinal section but conical and was screwed into a long-handled holder at an angle of around 70 degrees. The IDB was more effective than a conical rubber stimulator in terms of plaque removal, but did not reduce gingivitis. Subjects complained about the fragility of the metal wire and the possibility of damaging the gingiva. As in previous studies, there was a lack of specific information on the initial situation of the patients and on the selection and handling of the IDBs.

#### Situation from 1980 to 1990

In 1981, Bassiouny (Philadelphia, USA) and Grant (Manchester, England) presented a study on the cleaning of mesial and distal surfaces in 19 partially dentate individuals.^6^ In addition to dental sticks, they used IDBs (without providing further information on the product) with brush heads at an angle of approximately 70 degrees to the handle. IDBs cleaned the interproximal surfaces without adjacent teeth better than dental sticks, which in turn were more effective at cleaning the interproximal surfaces of teeth in contact. The authors cited the lack of interdental spaces that allowed the brush to penetrate as the reason for the poorer performance of IDBs. However, there was a lack of relevant information on the initial situation of the patients and the selection and handling of the IDBs.

In 1984, Bergenholtz (Umea, Sweden) and Olsson (Lulea, Sweden) compared the plaque-reducing effect of three different IDBs with waxed dental floss in nine subjects with ‘open interdental areas’.^8^ The IDBs were:

(a) the approximately 12 mm long ‘Interdental brush No. 2 dense’ from the Norwegian company Jordan with a cylindrical bristle field in longitudinal section and a ‘dense’ circular cross-section;
(b) the approximately 11 mm long ‘Short mini-interdental brush spaced’ from the Swedish company Brage Nilsson HB with a cylindrical bristle field in longitudinal section and a ‘spaced’ circular cross-section, which may have led to a lower resistance to insertion compared to point (a); and
(c) the approximately 18 mm long ‘Long mini-interdental brush spaced’ from the Swedish company Brage Nilsson HB with a cylindrical bristle field in longitudinal section as in (b) and a ‘spaced’ circular cross-section with correspondingly lower resistance to insertion.

The diameter of the twisted central metal core was specified as 0.71 mm for (a), 0.65 mm for (b) and 0.76 mm for (c). The outer diameter of all brushes was 6 mm. This means that the side bristles (filaments) had a length of just under 3 mm for all three products used.

At the beginning of the study, all test subjects underwent professional tooth cleaning. The various aids were each used for a period of 2 weeks. As a result, less plaque removal was observed with the use of dental floss than with the IDBs. The authors stated: ‘There was no difference in plaque removal between the three interdental brushes nor was there any difference in plaque removal on mesial and distal surfaces when interdental brushes were used. However, when dental floss was used, more plaque remained on mesial than on distal surfaces on molars and premolars. The difference was not as accentuated on incisors and canines as on molars [...] The gingival inflammation did not vary during the study. No lesions in soft or hard tissues were observed during the study. [...] Some patients claimed that the handles of the interdental brushes were too weak and often bent, which made the interdental cleaning procedure somewhat complicated.’ The authors continued: ‘It is important that the size of the interdental brush (both the brush and the metal wire diameter) match the interdental area, a fact which was neglected by Bassiouny and Grant^6^ and which explains why these authors found interdental brushes less effective than toothpicks and equal to ordinary toothbrushes in plaque removal.’ In conclusion, the authors wrote: ‘The reason for comparing the various interdental brushes was to analyse if the length and/or the density was of importance in plaque removal or in injecting the interdental area. The patients found all the interdental brushes easier to use and less time-consuming than dental floss. No difference in plaque removal or injury effect was found between the various interdental brushes.’

This means that a certain range of contact pressure was possible during the passage of an interdental space without this having a relevant effect on plaque reduction.

In 1987, Mauriello et al (North Carolina, USA) examined 15 subjects with large interdental spaces who had used IDBs (‘Butler Proxabrush’ from the American company Butler) for 3 weeks and compared them with other subjects who had used dental floss, a rubber stimulator or a conventional toothbrush alone.^53^ The use of IDBs as well as dental floss and a rubber tip stimulator led to a reduction in plaque compared to the use of a toothbrush alone. In contrast, there were no differences in the inflammatory parameters of the gums. The authors attributed the plaque reduction primarily to a Hawthorne effect (higher motivation of the test subjects when participating in a study). Also, there was a lack of relevant information about the initial situation of the patients and the selection and handling of the IDBs.

In 1988, Smith et al (Ann Arbor, MI, USA) presented a study on the effect of IDBs (John O. Butler, Chicago, IL, USA) in comparison to dental floss and rubber tip stimulators. They found no significant differences between the various products. Detailed information on the fit of the IDBs used in the interdental spaces examined was largely lacking.^71^

In 1990, Kocher et al (Kiel, Germany) investigated the cleaning effect of an IDB (Oral B, Procter & Gamble, Schwalbach am Taunus, Germany) under differently shaped pontics in 10 test subjects over a period of 9 weeks in comparison to brushing alone. They found – irrespective of the design of the pontics – a better plaque reduction after using IDBs.^44^

In 1990, Pöschke (Berlin Germany) presented a selection of the IDB ranges available at the time and named four manufacturers (Blend-a-med, Butler, Curaden and Oral B).^58^ The product range included brush designs with cylindrical or conical longitudinal sections. No details were given for the cross-sections. The central wires were either uncoated or nylon-coated. The sizes varied between ‘large’ and ‘small’ (without further quantitative details), whereby a colour code was used to differentiate between different sizes. The thickness of the central wire core ranged from 0.5 mm to 0.7 mm to 0.86 mm. The outer diameter of the brushes and the length of the side bristles were not specified. The wire cores had different buckling strengths, which were classified as either light or heavy. The brush heads had either free wire ends or plastic fastening systems. In particular, systems based on CPS (coloured plastic shank) were mentioned, in which the wire end was moulded with plastic as a retaining element. The brushes either had no separate holders or were equipped with holders or handles made of plastic or metal. No quantitative information was provided on the handle lengths. The positions of the brush heads in relation to the holders were described as either straight or angled, whereby the brushes presented were always angled. The author concluded: ‘Unfortunately, there is currently no complete system that can be used successfully and easily by inexperienced patients without restrictions and that can also be used in all situations.’

The ten publications published between 1970 and 1990 came from five countries (Norway, England, USA, Sweden, Germany). They prove that there was literature on the clinical effectiveness of IDBs, but provide no information on the level of awareness, access and dissemination in different countries around the world.

### Establishment of IDBs as Recognised Oral Hygiene Aids (From 1991)

This section provides the results of the reviews identified (Fig 1b).

#### Systematic reviews on the effectiveness of IDBs in a historical context

From 1991, several articles on IDBs were published. Nevertheless, it took some time before the first systematic reviews appeared. Figure 1b shows the process of the systematic literature search. Fifteen reviews relevant to the historical understanding of IDBs were identified and are discussed next.

In 2007, the Cochrane Collaboration presented the first systematic review of IDBs, which focused on their benefits for patients with fixed orthodontic appliances. However, the data were found to be insufficient to answer the benefit question.^30^ In 2008, Slot et al described the research landscape on IDBs as heterogeneous. In their systematic review, they concluded that IDBs, when used together with toothbrushes, significantly improve clinical parameters such as plaque scores, bleeding scores and probing depth compared to floss.^69^ Imai et al (2012) corroborated this by finding that the additional use of IDBs when brushing (compared to flossing) resulted in significant improvements in parameters such as plaque and bleeding scores.^37^

In 2013, the Cochrane database reassessed the topic of IDBs and came to the conclusion that there was still insufficient evidence to answer the question of whether IDBs reduce or increase plaque formation compared to dental floss.^57^ Despite a fundamental appreciation of IDBs, this was confirmed in the 2019 update.^56^ The latest update from the same year by Worthington et al also showed little evidence of a higher effectiveness of interdental brushes.^87^ A 2015 meta-review by Sälzer et al identified IDBs as the most effective means of interdental plaque removal. However, they found that the evidence for the combination of IDBs and toothbrushes in reducing plaque and gingivitis was only moderately strong.^62^ The European Federation of Periodontology workshop in 2015 also concluded that interdental brushes were the most effective for interproximal plaque removal, consistently outperforming both floss and dental sticks.^13^ In 2018, Kotsakis et al conducted a network meta-analysis on interproximal oral hygiene methods for reducing clinical indices of inflammation and concluded that IDBs and water jet devices ‘ranked high’ in reducing gingival bleeding, while flossing ranked last. However, due to the low power of the available studies, no definitive gold standard for interdental oral hygiene aids could be established.^45^

A 2019 study by Ng and Lim suggested that IDBs outperform brushing alone and are at least as effective (‘if not more effective’) than flossing in combating plaque and gingivitis. They also presented newer tools such as IRPs, which achieved comparable results to traditional IDBs.^55^ In the same year, Amarasena et al reported on a review of interdental hygiene devices, including flossing, IDBs, woods sticks and oral irrigation. They stated that the evidence for the effective-ness of these devices in managing plaque and gingivitis was weak to moderate and of low certainty and that there was no available evidence regarding their effectiveness in preventing dental caries.^3^ Gallie also shared this opinion in 2019, stating that the available evidence could not definitively confirm the effectiveness of interdental cleaning aids, including IDBs.^28^

Slot et al (2019) found that IDBs reduced plaque indices more than manual brushing. However, due to the lack of robust studies and the resulting uncertain evidence, they cautioned against drawing firm evidence-based conclusions about the effectiveness of any particular oral hygiene device.^70^ This statement was repeated by Shamsoddin in 2021.^68^

In 2022, Van der Weijden et al presented a systematic review in which IRPs were examined alongside IDBs and dental floss. They concluded that there is little difference in the treatment of gingivitis patients between IRPs, IDBs and flossing when used in conjunction with brushing. The study also showed that participants preferred IRPs, suggesting that they could be a viable alternative to interdental cleaning for these patients. However, they emphasised that longer-term studies are needed to determine their oral health benefits.^80^

If one considers the reviews as a whole, it is noticeable that the question of whether the subjects or patients used IDB sizes that were appropriate for the individual conditions of their interdental spaces was (and is) not dealt with very intensively. For example, almost all studies omitted information on the selected PHD values, which have been part of the ISO standard since 2006 (see next section).

#### Standards for IDBs

An ISO standard (16409) entitled ‘Dentistry – Oral hygiene products – Manual interdental brushes’ was first introduced in 2006. This was followed by a revised version in 2016.^38,39^

The following terms are defined:

(a) A manual IDB is a hand-operated instrument with filaments that emerge radially from a central wire to clean the interdental space.
(b) The brush head is the part that passes through the interdental space to clean accessible surfaces. It can be primarily fixed or removable. It is always fixed during use.
(c) The handle of the IDB holds its central wire.
(d) The core of the IDB is a twisted central wire, which in turn holds the filaments in place. It is either fixed in the handle or a connecting part or it fulfils the function of a handle itself.
(e) Filaments are individual bristles that are attached to the core.
(f) The core retention force is the force required to remove the shaft from the handle.
(g) The PHD is the minimum diameter through which a brush head can pass (using a clinically relevant force) without deforming the shaft.
(h) The brush size is an index of brush sizes that is determined by the PHD.

**Fig 13:**
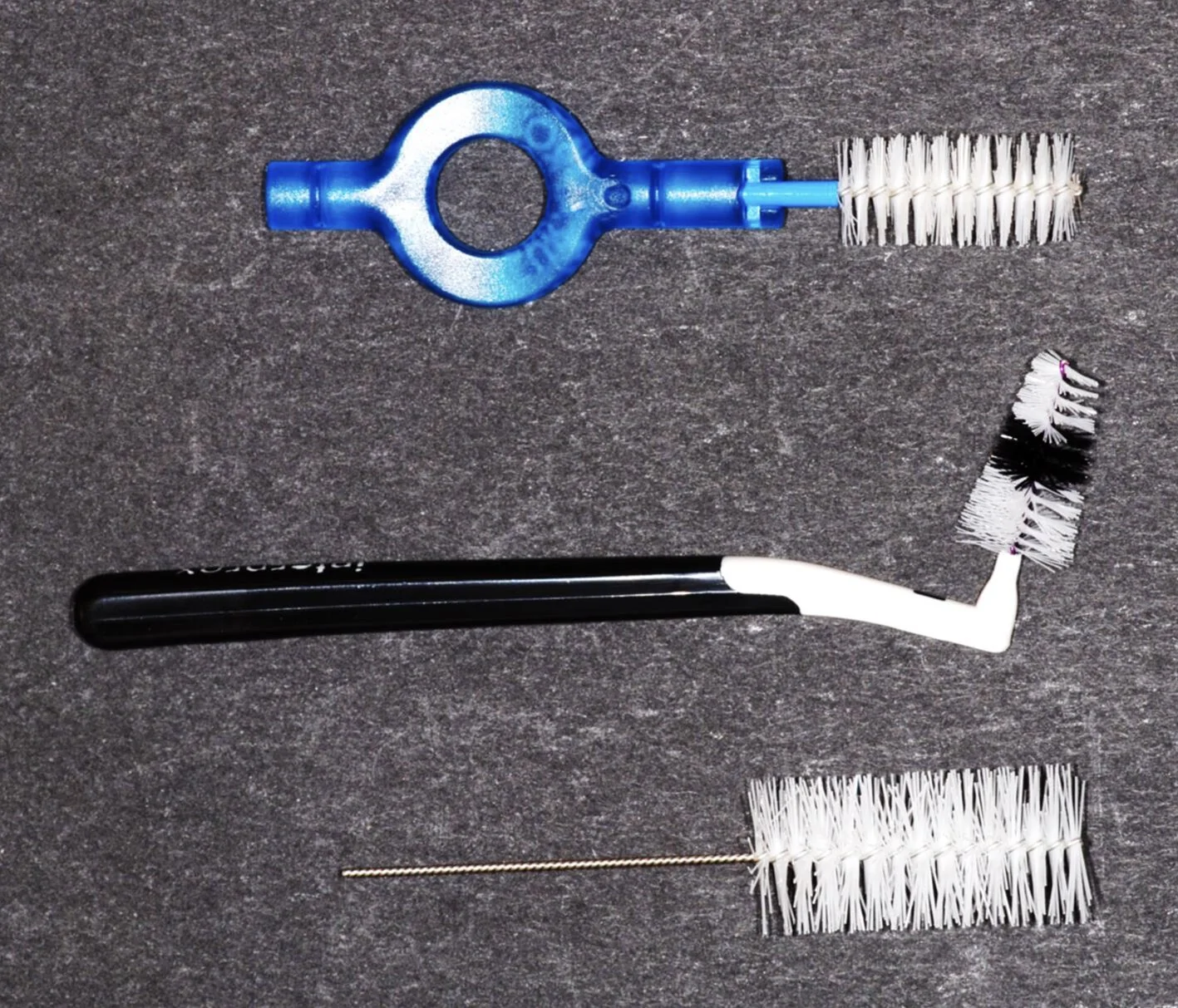
Manual interdental brushes, divided into three categories: *(a) *Type 1: with interchangeable heads that can be attached to a handle (Curaden, CH-Kriens). *(b) *Type 2: with permanent brush head (Dentaid, ES-Cerdanyiola).* (c) *Type 3: In this type, the wire core serves as a handle (Curaden, CH-Kriens).

Manual interdental brushes are divided into the following categories (see Fig 13):

Type 1: with interchangeable heads that can be attached to a handle.
Type 2: where the brush head is permanent.
Type 3: In this type, the core serves as a handle.

There are specific test requirements for IDBs, including the absence of defects, filament retention or core retention.

##### Passage hole diameter (PHD value)

**Fig 14:**
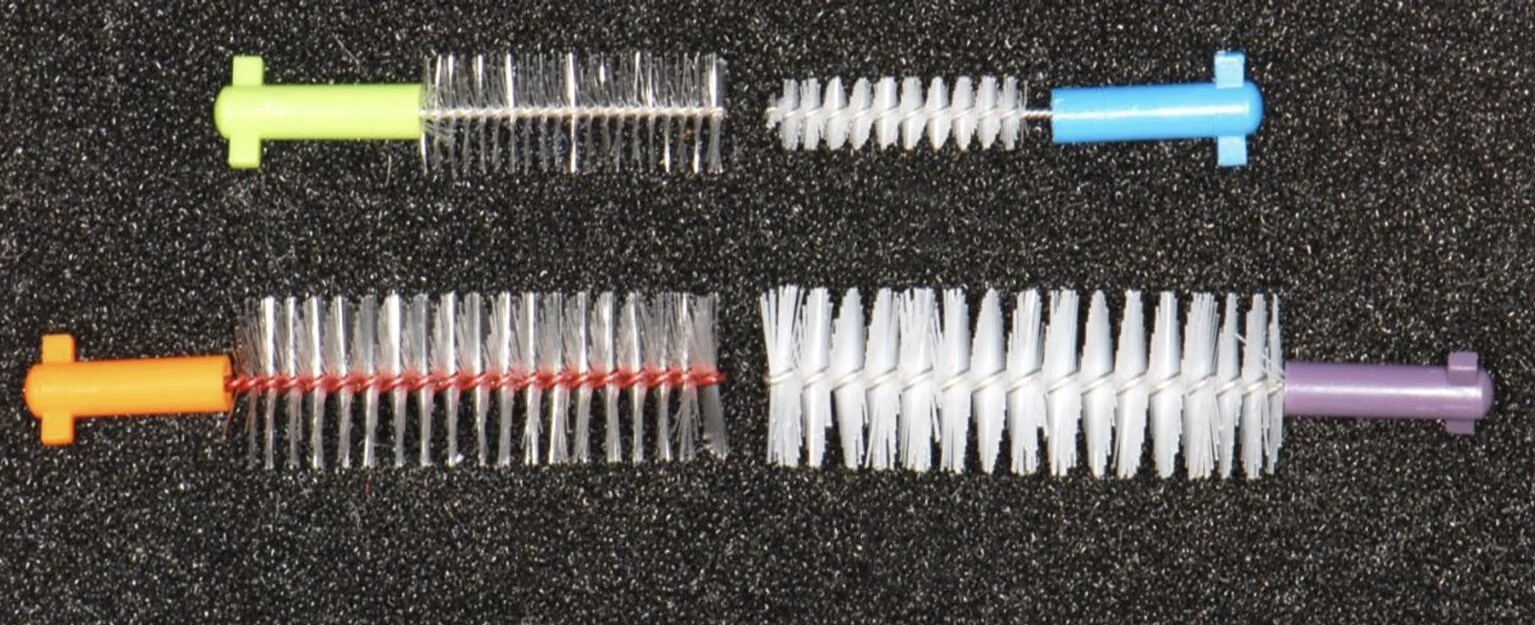
Determination of ‘sizes’ of IDBs with cylindrical outer diameter by visual inspection versus PHD value determination. *(a)* and *(b)*: the left interdental brush (green, CPS011, Curaden, Kriens, Switzerland) has an outer diameter of 5.0 mm. The right interdental brush (blue, CPS12), has an outer diameter of only 3.2 mm. Despite the significantly different outer diameter, they have the same PHD value of 1.1 mm.* (c)* and* (d)* the left interdental brush (orange, CPS507) has an outer diameter of 7.5 mm. The right interdental brush (purple, CPS18) has an outer diameter of almost 8.0 mm. Despite the similar outer diameter, they have significantly different PHD values of 1.9 mm (left) and 2.5 mm (right). The wire core is cylindrical in all types of IDBs.

In addition, there are ISO size standards for brushes based on the PHD value, which results from a complex summation effect of the properties of the central wire core and peripheral parts (filaments). Fig 14 shows some characteristics (wire core thickness, filament length and thickness) using the example of parallel IDB outer contours in longitudinal section.

Wire thickness, flexibility, coating and number of windings play an important role in the wire core. The filaments can differ in thickness, flexibility, arrangement, density and length, which is reflected, among other things, in the variability of the outer diameters in the longitudinal and cross-section. Such variability can also exist within a single brush.

**Fig 15:**
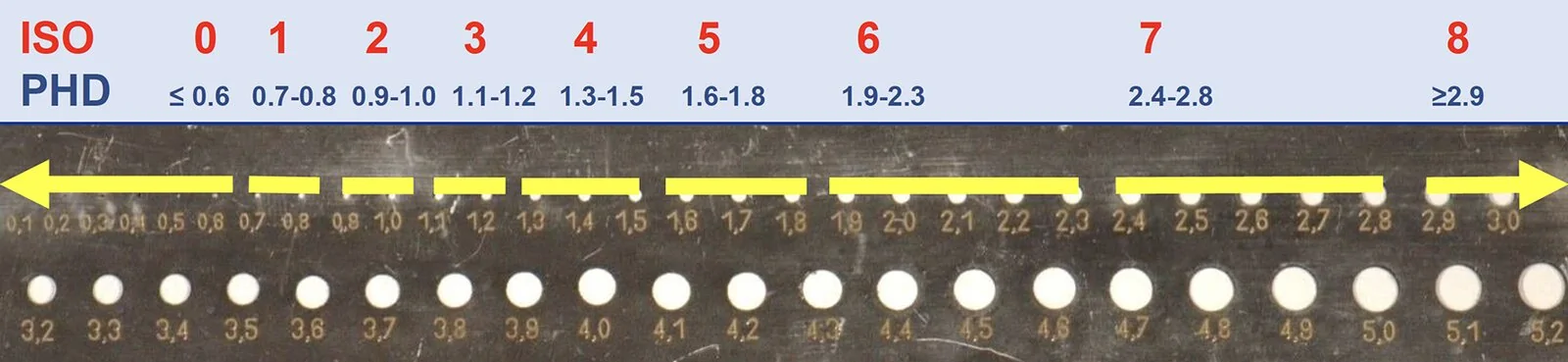
The ISO size specifications (0 to 8) are derived from PHD values but lack scientific validation. They are more or less arbitrarily chosen and can be misleading when selecting products. Therefore, it seems advantageous to assess the PHD values directly rather than the ISO sizes.

**Fig 16:**
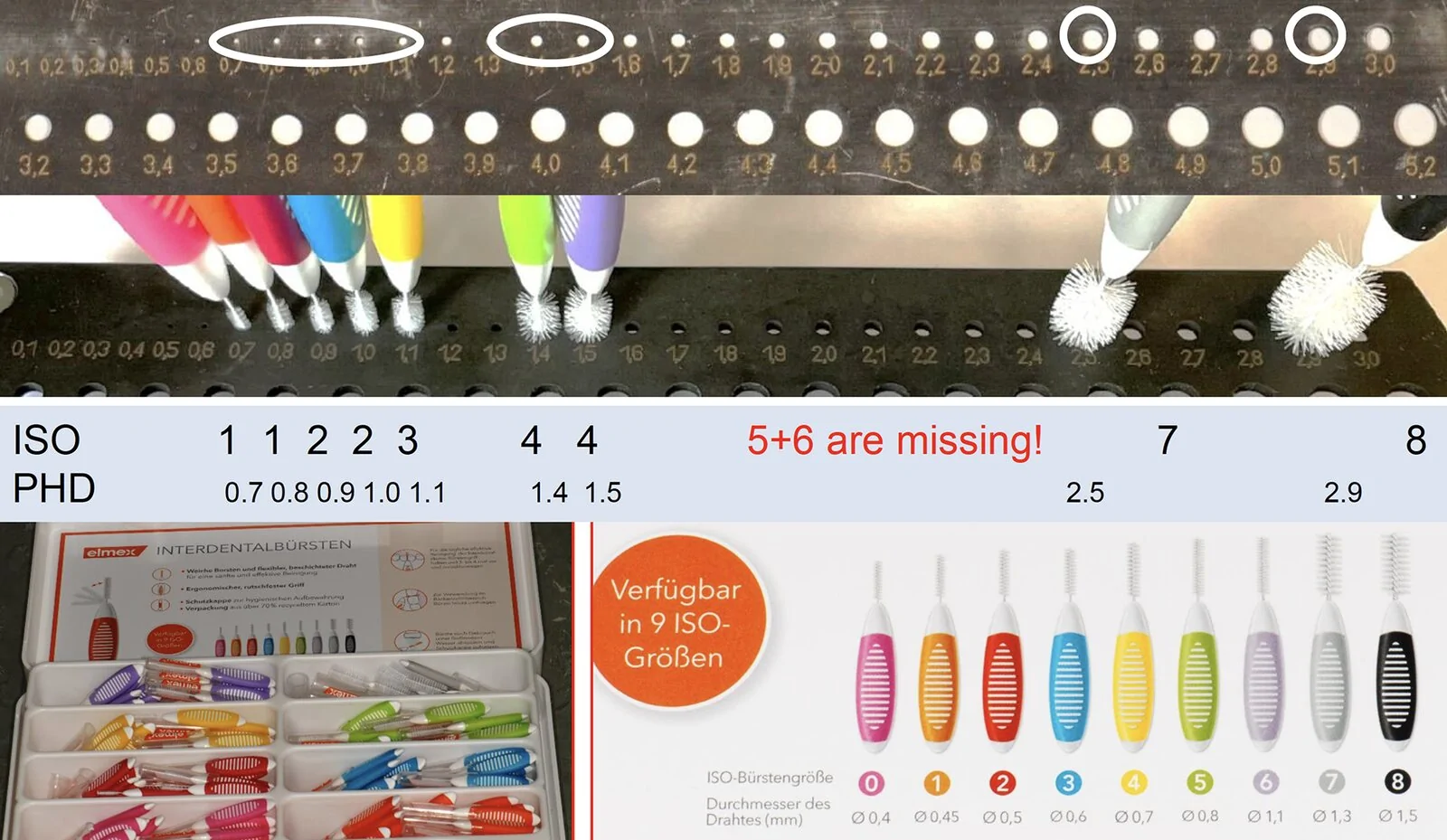
Example of a product system with discontinuous PHD values (here: Elmex, Gaba, Therwil, Switzerland). Top: Measuring plate with different hole diameters corresponding to PHD values. Middle: Measuring plate with equipped IDBs. Sizes 0.7 to 1.0 mm must therefore cover only a single PHD value, size 1.1 mm, however, covers three PHD values, size 1.4 a single PHD value, size 1.5 mm ten PHD values, size 2.5 mm four PHD values, and size 2.9 mm all PHD values from 2.9 mm. Bottom: According to the ISO size information presented in the brochure, the impression of a uniform distribution is created.

**Fig 17:**
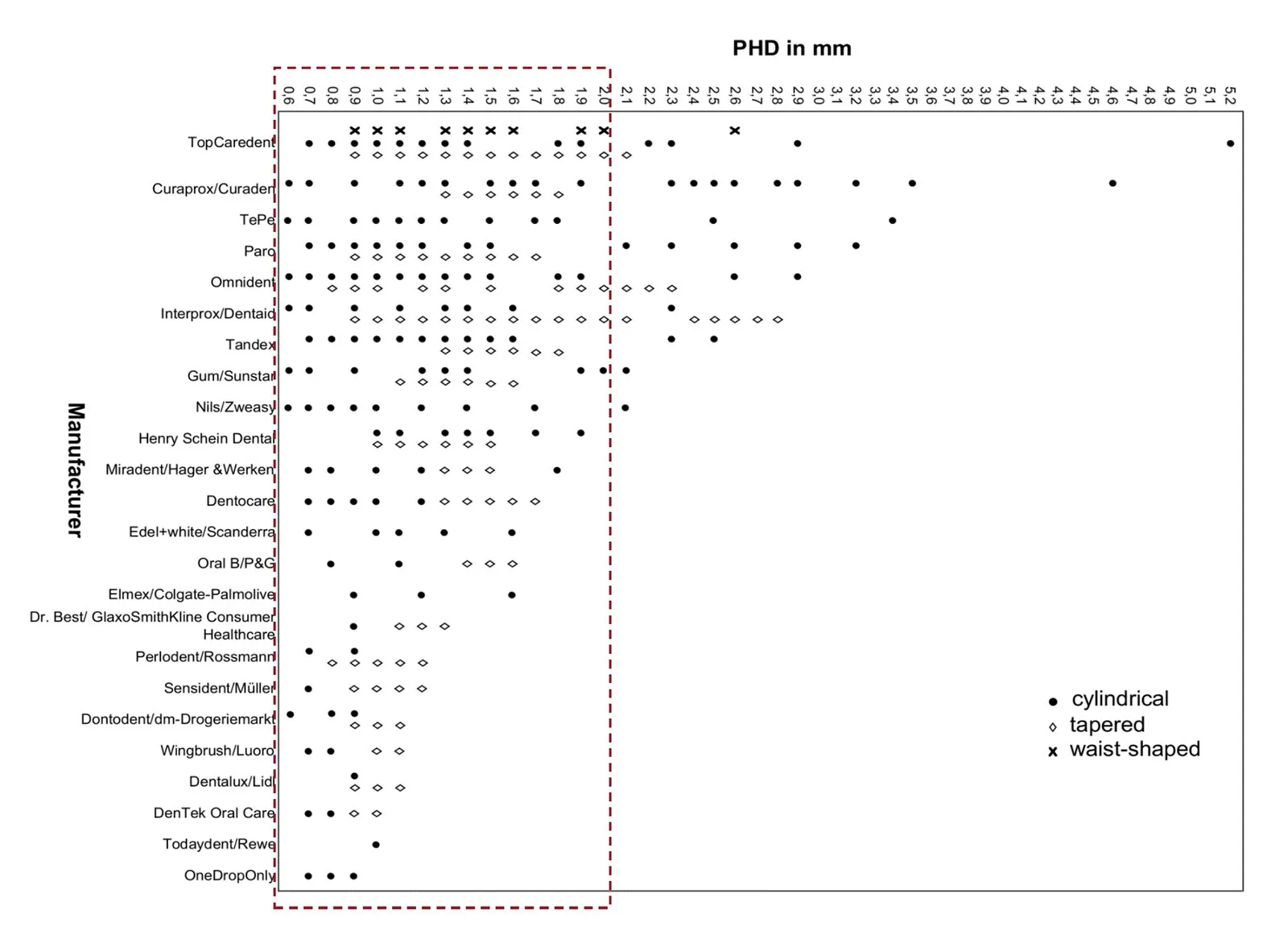
Spectrum of PHD values of commercially available interdental brushes (minimum value 0.6 mm, maximum value 5.2 mm) (source: Sekundo and Staehle, 2020 6^7^). It is noticeable that most products are small in size (low PHD), ie, where they are least needed. Patients with small interdental spaces can also clean their interdental spaces with dental floss, toothpicks, rubber picks, or just a toothbrush.

The ISO brush sizes are defined in Table 1. The intervals are not uniform and range from two PHD spectra for ISO sizes 1–3, three for sizes 4 and 5, five for sizes 6 and 7 to an undefined number for sizes 0 and 8 (Fig 15). Due to the different interval levels, a uniform distribution can be simulated, which, however, results in a clearly uneven distribution if the PHD values are taken as a basis (Fig 16). A study from 2020 revealed a PHD value spectrum of IDBs on the market ranging from 0.6 to 5.2 mm, with most IDBs concentrating on smaller sizes (Fig 17).^67^

**Table 1:** ISO brush size of interdental brushes

Size of the brush	Passage hole diameter (in mm)
0	≤ 0.6
1	0.7–0.8
2	0.9–1.0
3	1.1–1.2
4	1.3–1.5
5	1.6–1.8
6	1.9–2.3
7	2.4–2.8
8	≥ 2.9


The following sections of the standard contain information on test methods, test reports, additional information and packaging. The packaging must contain information on the manufacturer, the trade name and the brush size (PHD value).

#### Attributes and clinical application of IDBs

##### Selecting the size

Originally, the IDB sizes were determined on the basis of the specifications for wire core and total brush diameter in the cross-section.^82^ In some cases, the diameter of the individual bristles was also taken into account.^20^ An experimental study by Dörfer et al in 1997 showed that the cleaning effect of an IDB depends on its insertion resistance and the shape of the interdental space.^21^

An important reason for recommending IDBs is that evidence points to a better cleaning effect than flossing. It is expected that their side bristles will also reach into concavities that are merely spanned by dental floss. In the literature and in company brochures, corresponding illustrations are shown in which the ends of the IDB side bristles are at approximately a 90-degree angle to the central wire, reach vertically into the concavities and touch the tooth surfaces with their tips.^76^ However, Waerhaug already pointed out in 1976 that the lateral bristles position themselves parallel to the central wire or the tooth surfaces during a passage, which puts the above-mentioned ideas into perspective. Against this background, Waerhaug made it clear that IDBs can at best clean in wide furrows, but not in narrower concavities. Whether and to what extent IDBs can actually clean concavities of different shapes has not yet been systematically investigated clinically.

Despite these fundamental limitations, the proper selection of a suitable IDB is a prerequisite for successful application. While these are practical rather than evidence-based considerations, a brush that is too small may not adequately contact the surface and thus fail to remove sufficient plaque, and one that is too large could cause trauma to the gingival papilla.

Sizing based on PHD values was introduced in 2006 in accordance with the ISO standard (see previous section). However, many manufacturers continue to focus on wire and overall diameter. PHD values have not received much attention in clinical studies or in the selection of IDBs by the dental team, so patients are almost unaware of them. Against this background, selecting the appropriate IDB size remains a challenge.^16^ Determining the appropriate IDBs for a patient’s different interdental spaces is usually done by trial and error. Jackson et al advised starting with larger IDBs and moving to smaller ones if the initial sizes prove unsuitable.^40^

While the trial and error approach remains necessary, using PHD values can make the process more systematic. Instead of relying solely on visual judgement, which can be misleading due to variations between brands and appearances, the PHD value provides an objective starting point that can help narrow down the range of sizes to be tested. If the PHD value of an IDB that corresponds to the PHD value of the respective interdental space is used as a guide, the following procedure (known as the reference method) is recommended: After the clinical examination and determination of the probing depths, an initial impression is gained of the PHD values that are likely to be expected. If the first IDB used can already be inserted with a ‘clinically relevant force’ (according to the wording used in the ISO standard), the PHD value is correct. If the resistance is too low, the PHD value of the IDB must be increased in stages until resistance becomes apparent. If the resistance is too high, so that the selected IDB can only be inserted with great pressure or not at all, its PHD value must be gradually reduced until the desired resistance is achieved.^74^

To streamline this process, rigid, conical probes with a circular cross-section are available that are colour-coded for better visualisation (eg, IAP Curaprox, Kriens, Switzerland). Imai and Hatzimanolakis used these probes in a clinical study but did not comment on their suitability for IDB selection.^36^ Bourgeois et al^12^ investigated these probes in a highly selected sample of subjects with periodontal probing depths of less than 2 mm. They compared the penetration values obtained with the probes to the diameters selected by direct use of IDBs (reference technique) and concluded that larger diameters were generally obtained with the probes, which they referred to as the gold standard. However, it is uncertain whether these results are reproducible in patients with probing depths greater than 2 mm.

In practice, the values determined by probing can be far below the values found with the reference technique (see next). This is probably due to the fact that the rigid probes do not adapt as easily to the individual conditions of an interdental space as flexible IDBs. Further studies on these discrepant assessments are not available.

##### Filaments (side bristles) and wire core

The filament diameters can vary greatly.^20^ Some manufacturers differentiate between ‘original’ and ‘extra soft’ (TePe, Malmö, Sweden) or ‘regular’ and ‘prime’ (Curaprox, Kriens, Switzerland). A study by Wolff et al from 2006 showed that the individual bristle diameter had less influence on plaque removal than the overall diameter of the brush head.^85^ However, it can play a relevant role with regard to handling (easy or difficult to pass through) (see next).

The filament *lengths* of conventional IDBs, which are cylindrical in longitudinal section, can be calculated by subtracting the respective core diameter from the total diameter and then dividing this value by two. When an IDB passes through the interdental space with its wire core at the level of the gingival margin, the length of the side bristles (filaments) theoretically determines the potential for subgingival reach. For products currently on the market, this filament length ranges from approximately 1.1 to 7.0 mm.^75^ However, it is important to note that while longer filaments may have the potential to extend subgingivally, there is no evidence confirming the extent to which this actually occurs during use.

Little is known about the significance of the relationship between filament diameters and lengths. The longer side bristles are used, the more their diameter is likely to play a relevant role. It can be assumed that there are interactions between filament diameters and lengths with regard to both cleaning effectiveness and reach (shorter and thicker filaments with higher cleaning effectiveness and lower reach versus longer and thinner filaments with reduced cleaning effectiveness and higher reach) (see also Fig 14).

In this context, it has not yet been investigated from which filament thickness a cleaning effect can be developed at all. The idea that it is advantageous to combine very thin filaments with long filaments has not been proven and is also not plausible. Rather, it can be assumed that a favourable ratio of filament length and thickness must be used. However, systematic studies on this issue are not yet available.

The wire core can be either coated or uncoated. Although coating was widely discussed in the 1990s due to reasons of acceptance and a supposed risk of allergies, it ultimately failed to gain acceptance, particularly due to the unfavourable increase in the wire core diameter associated with it.

##### Brush head designs

**Longitudinal section:** IDBs in longitudinal section can have cylindrical, conical, waisted or mixed shapes. Rösing et al and Larsen et al found that both cylindrical and conical IDBs have a plaque-reducing effect, with conical IDBs being slightly inferior.^48,60^ Despite these results, one should refrain from recommending exclusively cylindrical shapes, as conical brushes may be more user-friendly for some patients. In 2023, Staehle et al presented a ‘compromise design’ that combines cylindrical and conical features (cylindrical cross-section with a slightly angled, conical tip).^75^

Chongcharoen et al observed better plaque removal with waisted longitudinal profiles than with cylindrical ones.^14^ Two sizes were used in their study. The waisted products had diameters of 5–3–5 mm and 7–4–7 mm, while the cylindrical products had diameters of 3 and 4 mm throughout. PHD value information was missing. For the patients with the waisted products, assignments of the two sizes were disclosed, but not for the cylindrical products, without a plausible reason being given. Against this background, it cannot be ruled out that the better plaque removal with the waisted brushes was also due to the size selection and not just the shape. In an *in-vitro* study, Baumgartner et al (2019) concluded that waisted brushes achieved a higher cleaning performance than cylindrical brushes with similar resistance to insertion.^7^ Schnabl et al (2019) confirmed this in an *in-vivo* study. They also found better plaque reduction with waisted IDBs than with cylindrical ones.^63,64^

The IDBs used were not labelled with PHD values by the manufacturer and were not examined by the authors in this regard. The authors did not provide information on which brush sizes were used, so that the dimensions of the IDBs compared cannot be assessed. However, they stated that they used such small sizes for the cylindrical IDBs that the test subjects were able to use the IDBs in the respective interdental space in both its mesial and distal parts (‘aspect of the space’). With the waisted IDBs, on the other hand, they used sizes that could only be placed in the ‘centre’ of the interdental space. This suggests that not only the lengths of the side bristles, but also the PHD values were different. It is therefore not entirely clear from this study whether an effect was achieved by the waisted shape or other parameters. Wehner et al (2021) found no differences in the cleaning effect between cylindrical and waisted brushes.^83^

None of the studies cited provided detailed information on the insertion resistance of the teeth or the PHD values of the IDBs used.

With regard to the passage forces, it is not only the longitudinal section design that is important, but also the filament thickness. For example, in order to reduce the initially higher insertion forces in cylindrical and tapered IDBs compared to conical IDBs, the anterior areas of the former can be equipped with thinner filaments than in the posterior area, so that the passage forces are equalised despite the different shapes.

**Cross-section:** In 1984, Bergenholtz and Olsson investigated modified circular cross-sections of IDBs8 (see ‘Situation from 1980 to 1990’). In one case, the brush field was arranged more densely (‘dense’), in the other case more loosely (‘space’), which may have led to a lower resistance to insertion. The side lengths of the bristles (just under 3 mm) were identical for both products. There were no differences in the extent of plaque removal, which indicates that the length of the side bristles was more relevant under the given test conditions than a slight difference in the insertion resistance of the overall brush.

**Fig 18:**
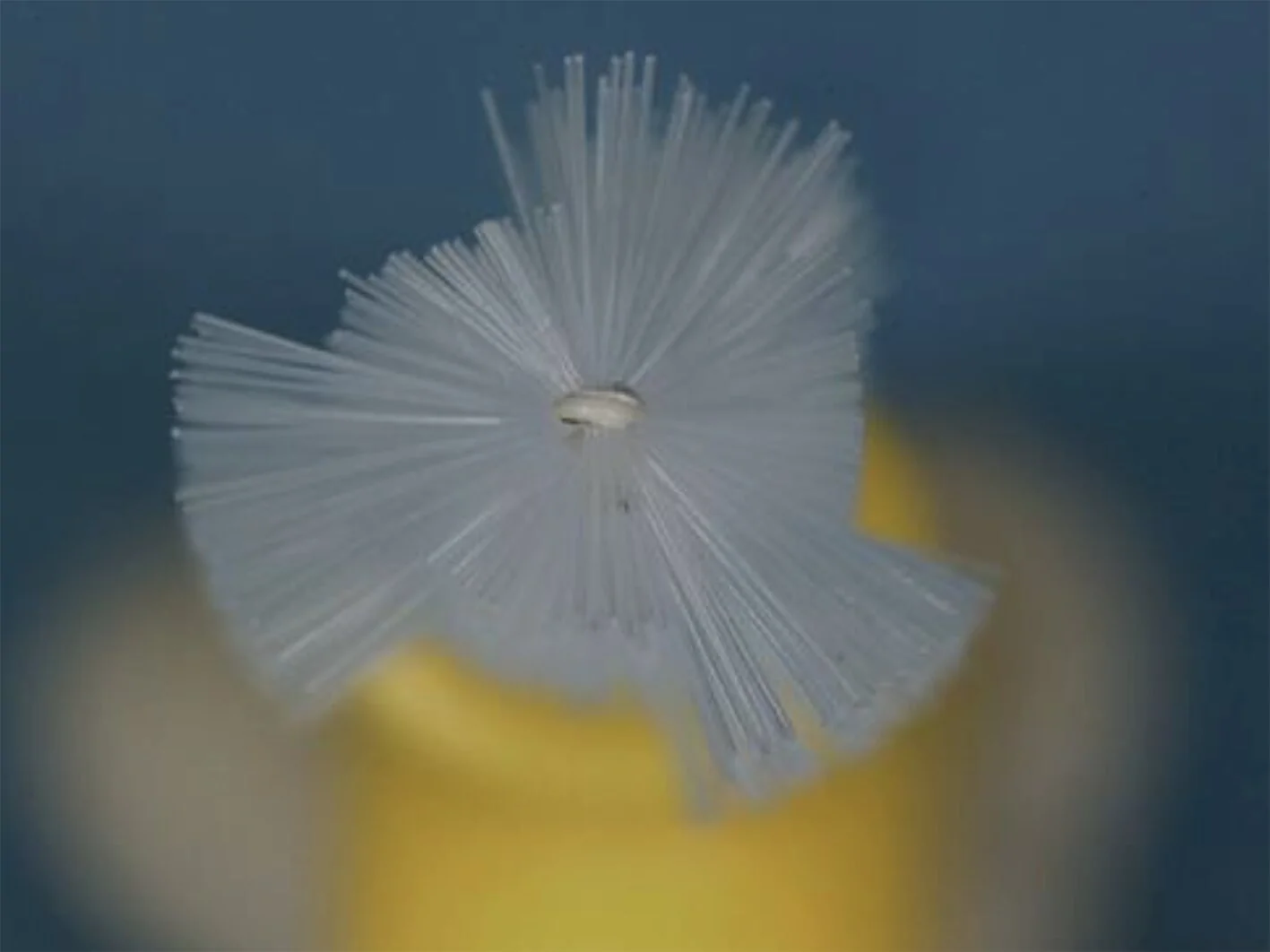
Interdental brush with a triangular cross-section (source: Wolff et al, 2006^84^).

In 2006, Wolff et al found that triangular cross-sections reduced resistance while maintaining plaque removal efficacy (Fig 18). However, their *in-vitro* model did not correspond to the actual positioning of the wire cores *in vivo*.84 While they are positioned centrally *in vitro*, they are positioned at the level of the gingival margin *in vivo*. However, studies on this are not known.

**Fig 19:**
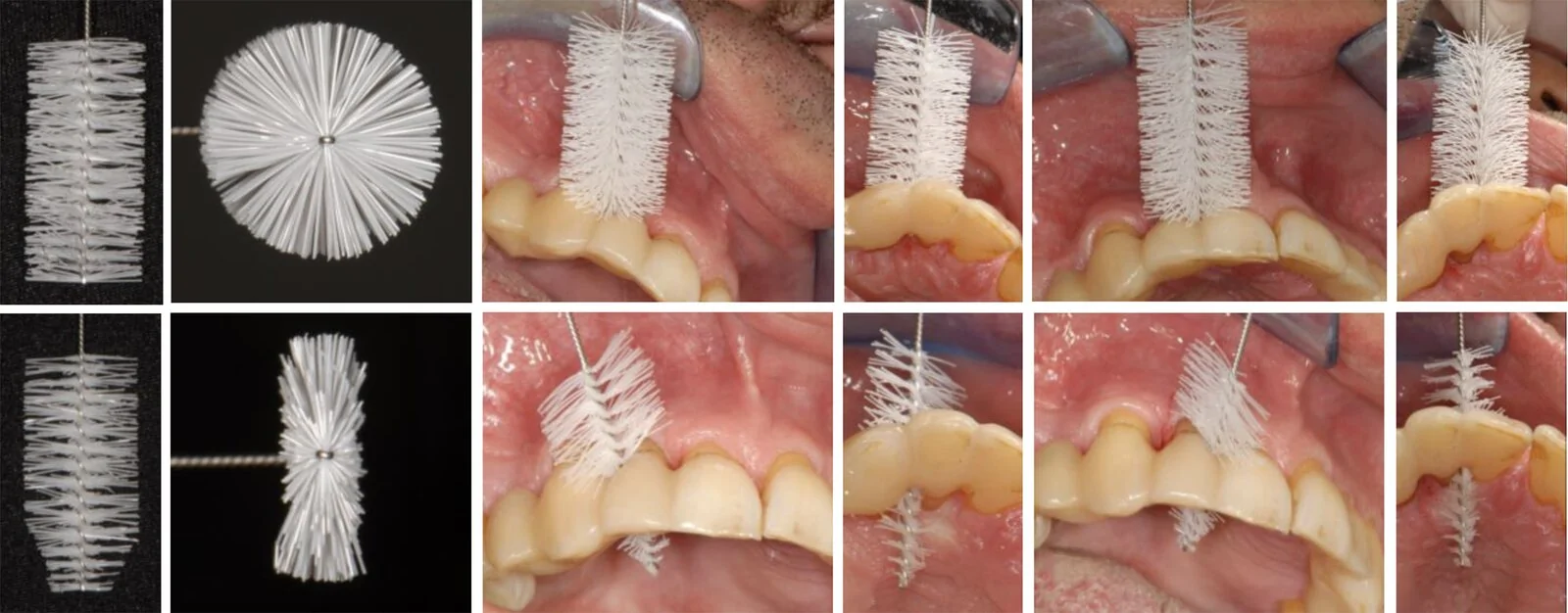
Fitting of IDBs with side bristles (filaments) of 7 mm length (LS 637, Curaden, Kriens, Switzerland) in the interdental spaces of a 49-year-old patient with pocket depths of 6 mm each at the upper right lateral incisor distally and mesially. *(a)* and *(b) *IDB with cylindrical longitudinal section and circular cross-section (PHD value: 4.2 mm); *(c)–(f) *no insertion possible;* (g) *and* (h)* with a tapered tip of the cylindrical longitudinal section and a flattened cross-section (thereby reducing the PHD value to 2.5 mm). *(i)–(l) *insertion now possible.

In 2023, Staehle et al introduced flattened IDBs for the first time (Fig 19), and demonstrated their effect on reducing PHD values compared to circular cross-sectional shapes. In the case report, the use of flat brushes resulted in stippled gingival surfaces, which indicate increased keratinisation, reduced probing depths, and an absence of bleeding on probing.^75^ These observations led to the hypothesis that these design changes could be of clinical relevance. However, this serves only as a foundation for further studies, such as randomised controlled trials, to assess the positive or negative effects of these design modifications on a larger case group. No further studies are available to date on the clinical use of flattened interdental space brushes.

The following considerations should be considered with regard to the anatomical structures affected: if subgingival areas are slightly extended, the interdental space brushes are likely to move between root surfaces and coronally located gingival tissue. Areas that extend further subgingivally are more difficult to access. It is not known exactly what type of tissue the flexible side bristles (ends) of an interdental brush come into contact with.

In the vertical passage of rigid periodontal probes, it is assumed that in the case of healthy periodontal conditions, the probe tip comes to a standstill approximately 0.4 mm coronal to the apical end of the junctional epithelium. In the case of periodontal inflammation with pathologically altered pocket epithelium, however, the probe tip penetrates the inflamed tissues at a probing depth of up to 5.5 mm (PSI grade 3), for example.^24,49^

The mechanisms by which mechanical plaque removal affects inflammation may be multifaceted, ranging from cleaning and loosening of plaque to the disruption of microflora through mechanical irritation and influencing the biofilm by altering the local ecology. However, it seems plausible that reaching deeper areas on exposed subgingival tooth surfaces will be beneficial, regardless of the individual morphological situation. To date, the subgingival effect of interdental cleaning is not known.

##### Holder designs (straight or angled)

A 2014 study by Jordan et al found that straight IDB retainers were more effective at removing plaque in the posterior region compared with angled retainers.^41^ In the posterior region, they found a higher plaque reduction with straight holders than with angled holders. Possible reasons for these findings were not given. The sizes of the IDBs were determined using IAP samples (see above). It is not clear from the test description whether a test subject used the same IDB size for all their interdental spaces or whether several IDBs were used, taking into account the individually different interdental spaces. PHD values were not stated. It is possible that smaller IDBs with angled holders, which allowed vertical positioning, had a lower resistance to insertion than IDBs with straight holders, which were more likely to cause wedging due to the narrow local conditions in the posterior region. Studies conducted under comparable conditions with recording of the PHD values per interdental space are not available.

##### IDB wear and tear

Even in the early years of IDBs, there were isolated complaints about signs of wear such as wire kinking or bristle loss.^8,54^ The ISO standard sets a minimum standard for durability.^39^ However, to date there have been no studies that systematically investigate signs of wear. A reduction in wear and the resulting longer durability would not only meet the needs of patients, but also ecological requirements.^1^ In addition, evaluating the wear behaviour of IDBs could not only provide indications for improvements, but also information about bristle positions during clinical use, which is relevant for understanding their mode of action.

##### IDB assortments

**Fig 20:**
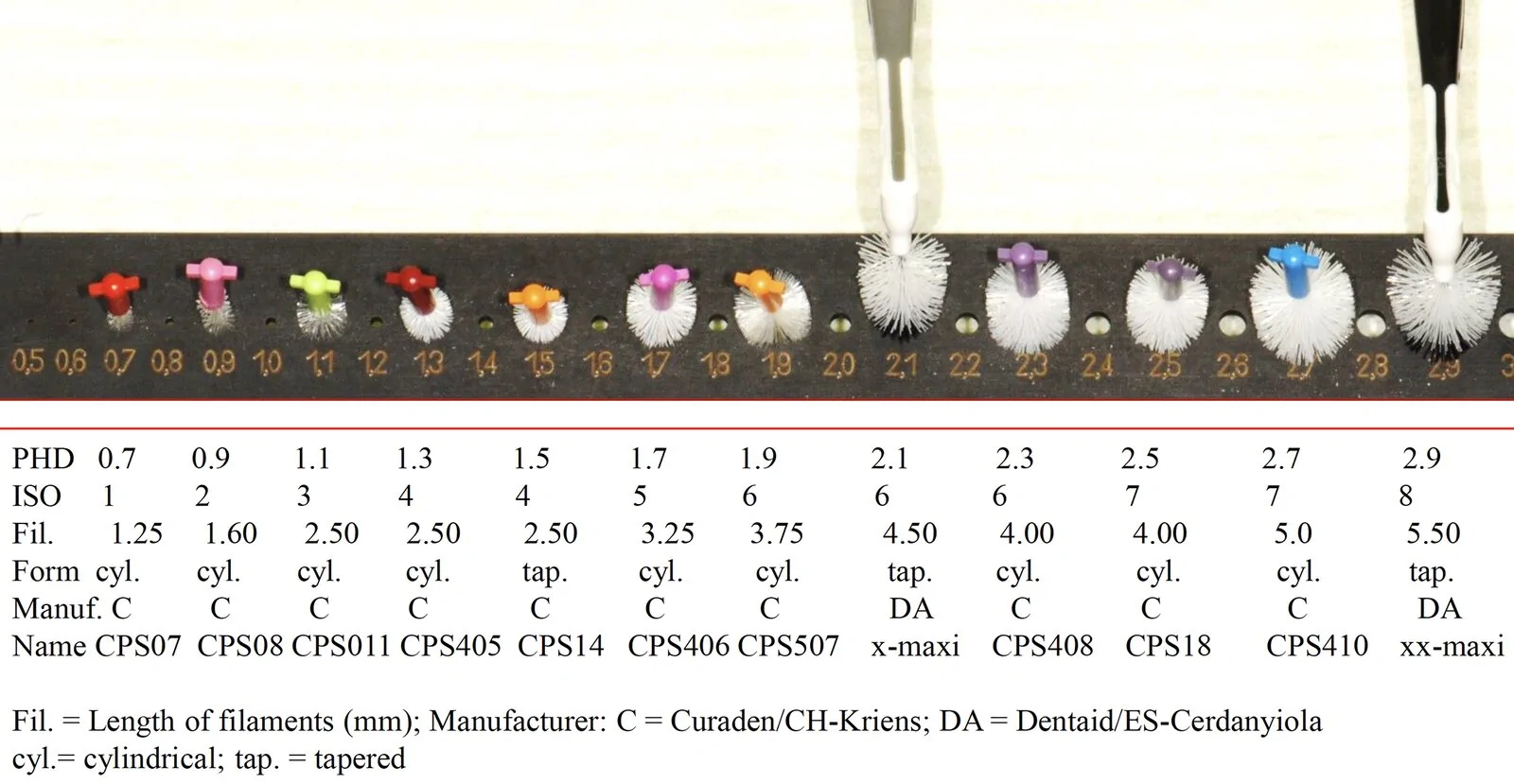
Example of a product system with a uniform distribution (here: various manufacturers). Selection of 12 conventional interdental brushes (circular cross-section) with continuously increasing PHD values in steps of two (here: 0.7 mm to 2.9 mm) (‘Heidelberg Set’). The numerical values can vary by about 1 to 2 PHD values depending on the force applied during insertion (especially with thicker IDBs). With conical shapes, the initial applied force is lower. In addition to the PHD values, the lengths of the side bristles are indicated (Staehle et al., 2021^74^).

Since the 1980s, the product range has expanded, allowing IDBs to be organised into assortments. A 1998 publication by Christou et al presented a range of six IDBs from Enta-Lactona BV in Bergen, the Netherlands.^15^ These brushes had a steadily increasing outer diameter of 2.5–8 mm, which means a side bristle or filament length of about 0.8 mm to 3.5 mm. Currently, some manufacturers offer IDB series tailored to specific purposes and target groups, eg, young and healthy patients, implant wearers or periodontitis patients. However, there is often a lack of scientific evidence for these selections. Other manufacturers stick to ISO size groups, which imply an evenly distributed range, but this is not always the case. A new range was therefore introduced in 2021 with the aim of offering a selection with evenly increasing sizes based on PHD values for patients of all ages. The range consists of 12 IDBs in evenly ascending order, each separated by two PHD intervals, and covered a wide range from a PHD value of 0.7 mm to 2.974 (Fig 20). It reflects the status quo of 2021, but still requires further scientific validation and any necessary adjustments.

**Fig 21:**
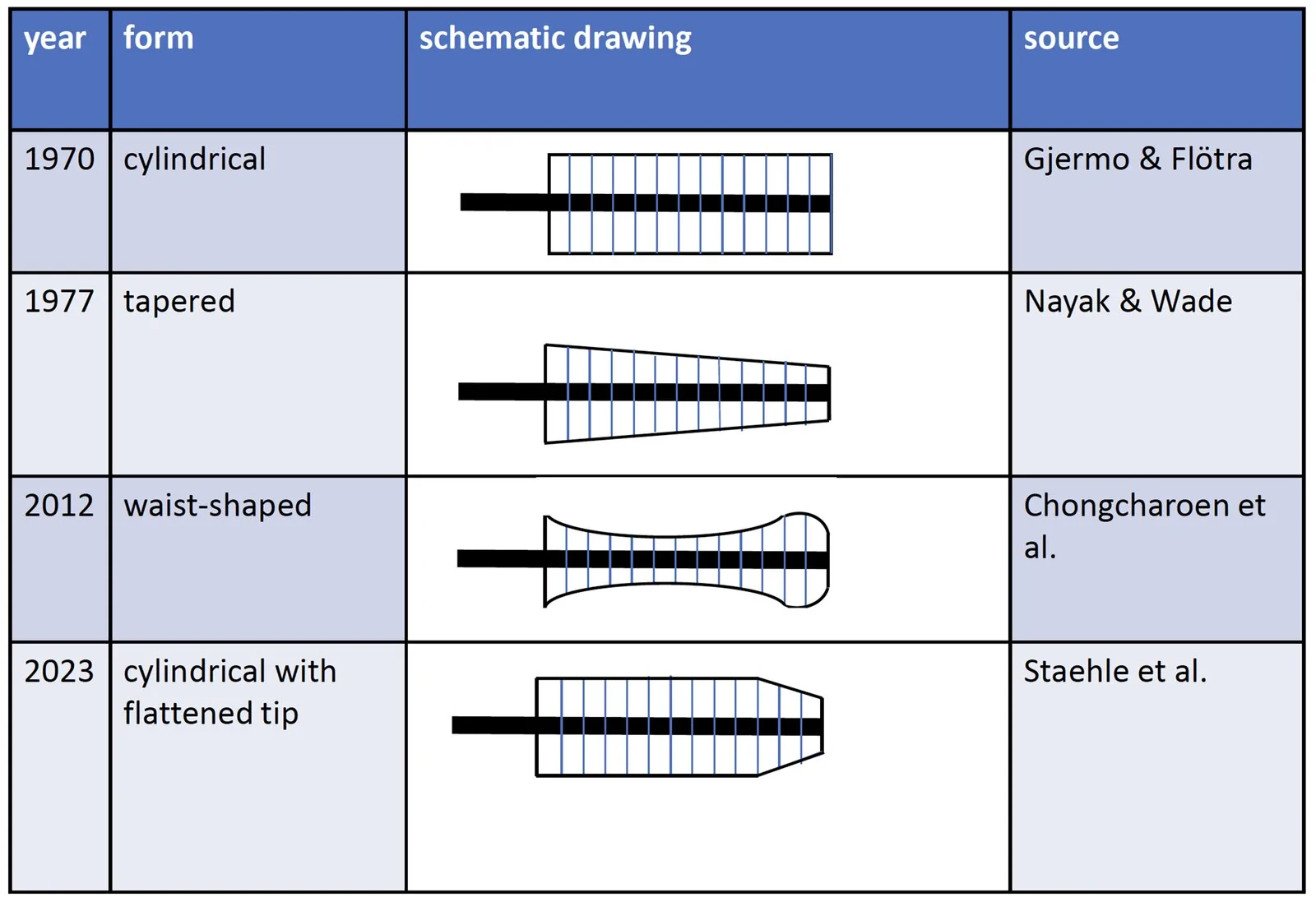
Historical developments of IDB designs in longitudinal section.

**Fig 22:**
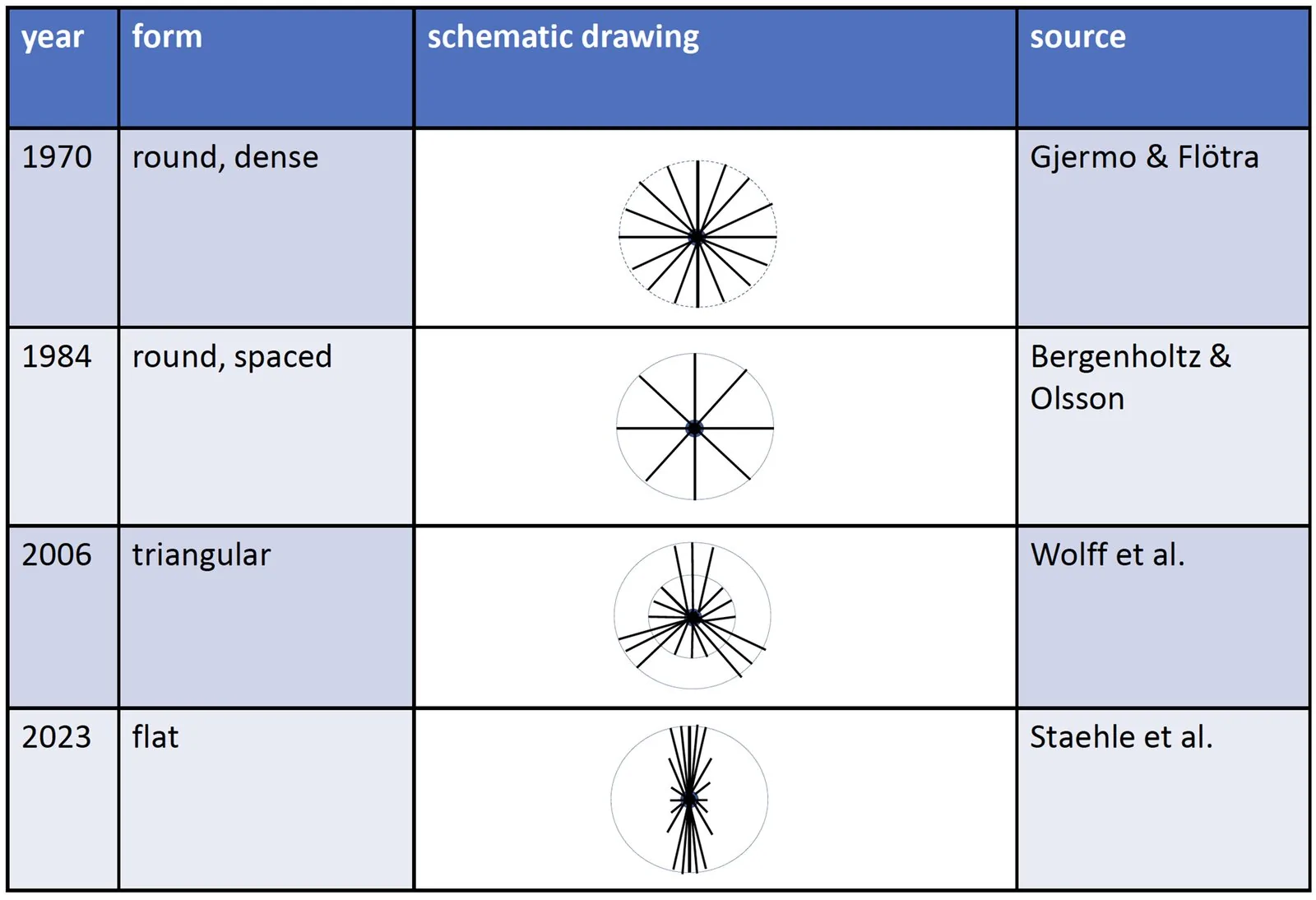
Historical developments of IDB designs in cross-section.

**Fig 23:**
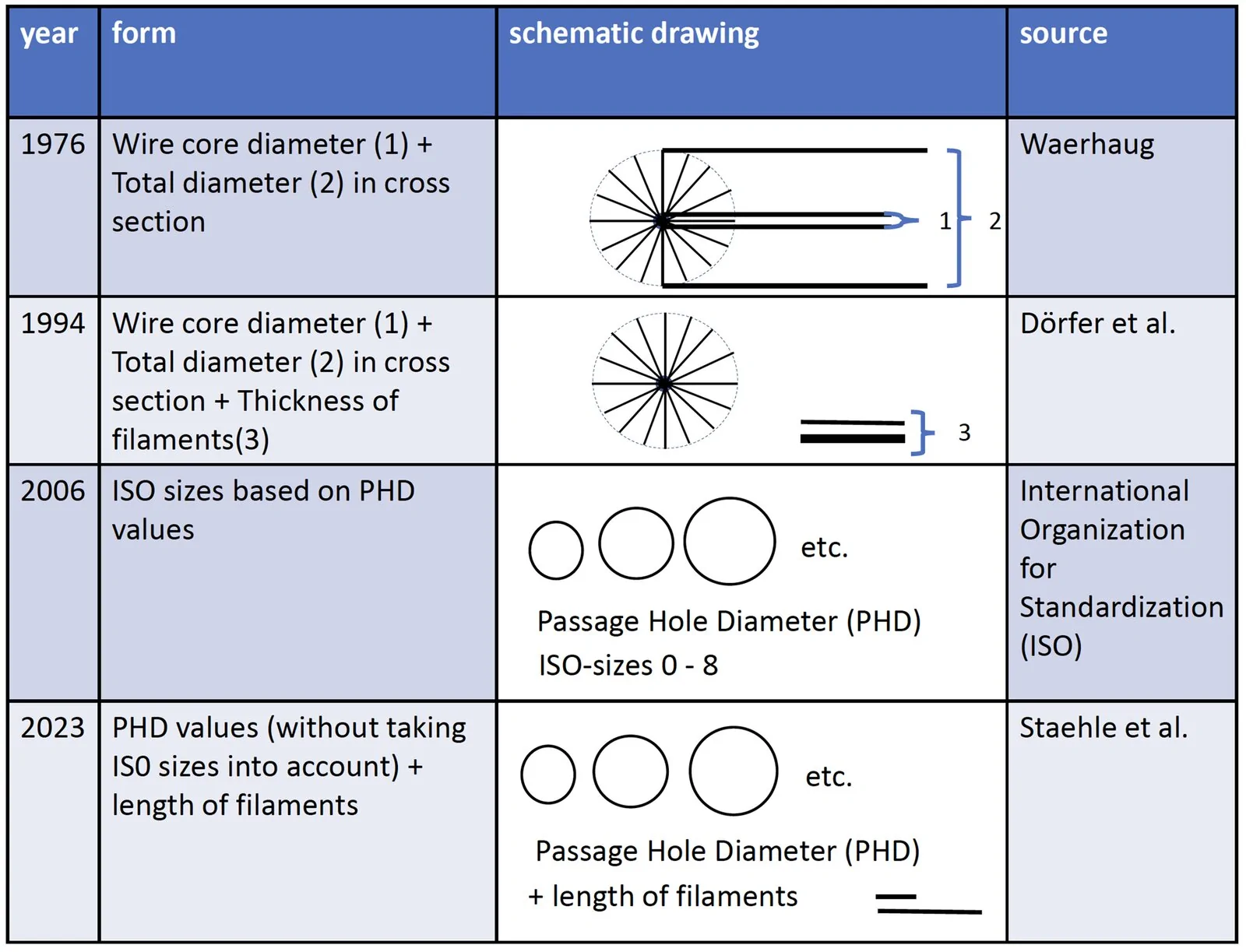
Historical developments in the declaration and classification of IDBs.

**Fig 24:**
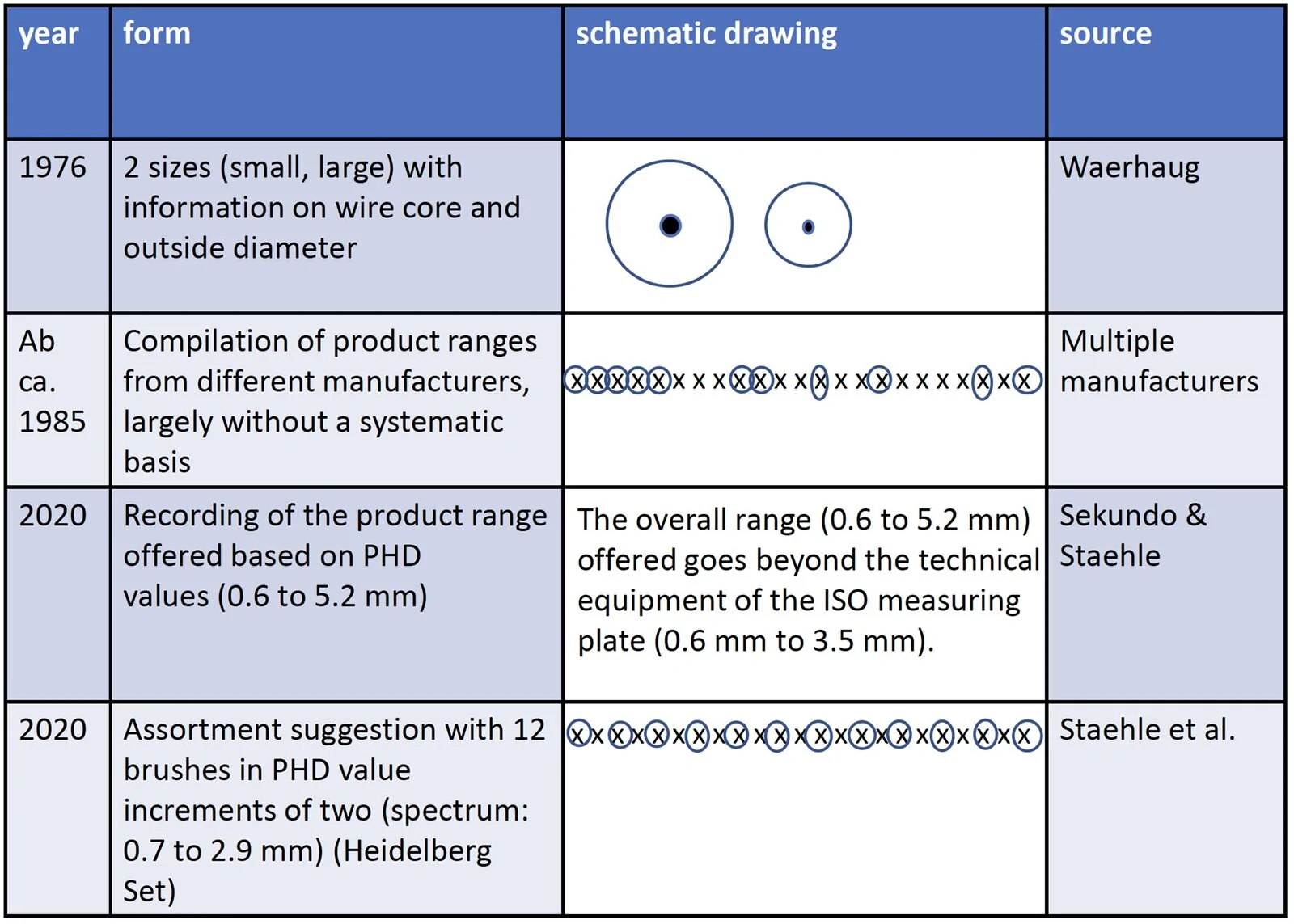
Historical developments of IDB assortments with available spectra.

The development of the designs and product ranges over the years is shown in Figures 21 to 24. The product range profile was further developed in 2024 by Sekundo et al.^66^

##### Risks from IDBs

When using IDBs, the filaments and/or wire cores may traumatise the tooth structure and the surrounding soft tissue.

In 1991, Reiter and Wetzel found that the bristle ends of the IDBs were not sufficiently rounded.^59^ Dörfer et al also pointed out possible injury risks in 1994, which can be caused by the quality of the bristle ends and the wire contours, among other things.^20^ The extent to which the bristle tips of IDBs round off naturally during use remains undetermined. Due to the possibility of damage to healthy interdental soft tissue, a risk-benefit assessment must be carried out before recommending use, which requires a dental examination.

Regarding the potential damage to hard tissue, Dörfer and Staehle (1998)^22^ and Eickholz (2021)^24^ showed individual cases in which interproximal cervical defects were observed, which raised the question of a possible connection with the use of IDBs in combination with toothpastes. However, they were unable to provide more detailed information about the history, so that causal relationships between IDB use and tooth structure damage could not be established. The lack of scientific studies taking into account various influencing factors suggests that such damage has not been widely recognised in the past.

With regard to damage to soft tissue, Waerhaug (1976) and Bergenholtz (1984) found no correlation with the use of IDBs.^8,81,82^ In 1984, Badersten et al stated that IDBs could exert mechanical pressure on the interdental papilla, possibly leading to recession of the marginal gingiva.^5^ Jackson et al (2006), on the other hand, argued that the depression of the interdental papilla by IDBs was only temporary. They suggested curving the central wire to avoid potential injury, especially lingually and palatally.^40^ According to their observations, shrinkage and recession of the papillae after IDB application were a healing response due to swelling and oedema reduction rather than adverse effects. Tu et al (2008) also emphasised that a decrease in probing depths with IDB application was due to a reduction in inflammation (indirect effect) rather than permanent papilla compression (direct effect).^77^ The Danser index (DI) was developed as a method for determining gingival abrasion in order to assess the sensitivity of the gingiva to oral hygiene products.^18^ Jordan et al (2014) found no abrasion of the gingiva when using IDBs.^41^ In contrast, Hennequin-Hoenderdos et al found greater gingival abrasion with IDBs compared to rubber picks.^34^

##### IDBs compared to IRPs

As interdental rubber picks are receiving more and more attention in the literature, they will be discussed in more detail here, also to differentiate them from IDBs.

**Fig 25:**
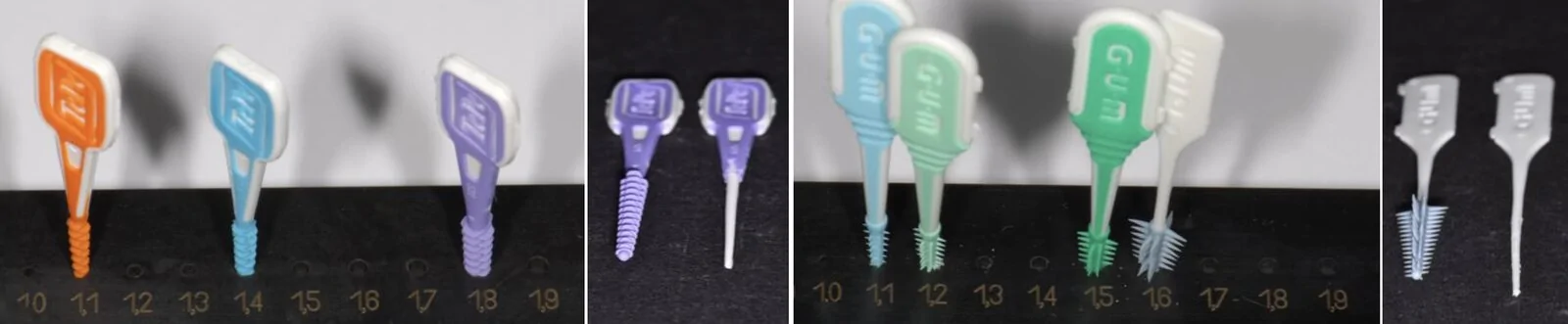
*(a)* The Easy Picks from TePe (Malmö, Sweden) are available in three sizes. Size small (XS/S, orange) ranges from a PHD of 0.8–1.4 mm (middle position PHD 1.1 mm); size medium (M/L, blue) ranges from a PHD of 1.1–1.7 mm (middle position PHD 1.4 mm); size large (XL, violet) ranges from a PHD of 1.4–2.1 mm (middle position PHD 1.8 mm). *(b) *Representation of the XL shape with (left) and without (right) sheath. The conical core (right) has a diameter of about 1.2 to 2 mm, the lateral ridges or lamellae (left) have a length of about 0.4 to 0.8 mm. *(c) *The Soft-Picks from Sunstar GUM (Etoy, Switzerland) are available in four sizes. Size small (S, blue) ranges from a PHD of 0.8–1.4 mm (middle position PHD 1.1 mm); size medium (M, light green) ranges from a PHD of 0.9–1.5 mm (middle position PHD 1.2 mm); size large (L, green) ranges from a PHD of 1.0–1.6 mm (middle position PHD 1.5 mm). Size extra large (XL, grey) ranges from a PHD of 1.3–2.1 mm (middle position PHD 1.6 mm).* (d) *Representation of the XL shape with (left) and without (right) sheath. The conical core has a diameter of about 1.1 to 1.8 mm, the lateral thorns have a length of about 0.7 to 2.5 mm. The taper of the oral hygiene aids shown here is achieved not only by the outer sheaths (lamellae or thorns) but also by the conical plastic wire cores (combination effect). In contrast, the wire cores of IDBs are always cylindrical, and taper is achieved exclusively by the filament lengths (see Fig 13).

In contrast to IDBs, IRPs do not have a metal core or nylon filaments, but a plastic core with short lamellae or rubber spikes or ‘silicone flaps’. IRPs are also referred to as rubber interdental bristles (RIBs) or rubber bristles interdental cleaners (RBICs). Some sizes and shapes are illustrated in Figure 25. While the wire core of IDBs is always cylindrical and outline contours can therefore only be changed using filaments of different lengths, both the core and the outer parts of RPs can be varied. This allows a much greater design freedom with the effect that only comparatively few sizes are required to cover a larger PHD spectrum.

In 2014, Abouassi et al compared such new rubber cleaners with IDBs and concluded that bleeding on probing could be reduced with both oral hygiene aids, although the study participants found the metal-free rubber cleaners to be more pleasant. This study is also worth mentioning because the instruments used were characterised with PHD values, which facilitated an objective comparison. The authors came to the following conclusion: that RIBs can be used as an alternative interdental cleaning product which may be more accepted by patients.^2^

In 2018, Hennequin-Hoenderdos et al compared an RBIC with an IDB and found better results with the RBIC than with the IDB in terms of the reduction of bleeding on probing and plaque, tissue sparing and patient acceptance.^34^ They made the following conclusion: In accessible sites, the RBIC in conjunction with manual toothbrushing, was found to be more effective in reducing gingival inflammation after 4 weeks. The RBIC caused less abrasion of the gingiva and was appreciated more by the participants. The decision for an IDB or an RBIC depends on the individual patient’s conditions, including the size of the interdental space and the degree of gingival inflammation.^34^

In 2022, Graetz et al used various study designs to realistically test the interdental rubber picks *in vitro*. They found that the presence of artificial saliva alone reduced the cleaning effort by half and allowed larger diameters of the cleaning instruments used, which considerably increased cleaning effectiveness and patient acceptance.^32^

In the previously cited review by Van der Weijden from 2022 (see earlier), reference was made to the need to generate studies that provide information on long-term effects.^80^ There are also hardly any studies to date on correct size selection, positioning during passages, handling or signs of wear.

##### Instructions on the handling of aids for interdental cleaning

Instruction on interdental cleaning is usually given by dental assistants or dental hygienists. In a study on instruction practice, it was found that dental professionals sometimes do not follow official recommendations from professional societies, but pursue their own preferences, considering patient wishes.^33^ When asked about their own interdental oral hygiene practices and their patient recommendations, the following responses were given: Dental floss (83.5% and 78.0%, respectively), IDBs (77.1% and 95.4%, respectively), dental sticks (0.9% and 0.0%, respectively), rubber picks (15.6% and 26.6%, respectively) and oral irrigators (0.9% and 5.5%, respectively). 16.5% of respondents combined the use of IDBs with the use of toothpastes as part of their own oral hygiene. They also recommended this procedure to 22.0% of their patients.

The authors pointed out that this was not in line with the guidelines of professional associations, which, for instance, advise against using toothpaste with IDBs. The reason given for this recommendation is the risk of increased destruction of the interdental tooth structure as a result of the abrasive agents contained in toothpastes.^33^ Broader evidence regarding oral hygiene practices, such as the use of toothpaste with toothbrushes, suggests that toothpaste does not enhance the mechanical cleaning effect compared to using toothbrushes without toothpaste.^78^ Therefore, the use of toothpaste with IDBs is unlikely to offer additional benefits. Given that the potential risks, even if rare, could have relatively serious consequences, these guidelines err on the side of caution. However, it must be noted that this recommendation is based on limited clinical observations and lacks strong evidence.

This example illustrates the challenges of instructing patients on oral hygiene practices. There is a lack of evidence for many recommendations concerning the selection and use of oral hygiene aids. Consequently, providing appropriate instructions and advice are only possible to a limited extent.

## DISCUSSION

From on-site research and the literature analysed, it appears that IDBs have probably been in use since the first half of the 20th century (since 1960 at the latest), with scientific studies on their handling and effectiveness beginning in 1970. Originally, IDBs mainly had a cylindrical design in the longitudinal section and merged without angulation into the coiled double wire, which also served as a handle. Later, other shapes, such as conical, were also offered. IDBs were circular in cross-section, and designs with dense or loose filaments were available. Additionally, attachments were developed in holders aligned at approximately a 70-degree angle to the handle.

As early as 1976, it was determined that the side bristle and filament lengths of IDBs influence the depth of subgingival plaque removal. The filament lengths of the IDBs available at the time (around 3 mm) resulted in subgingival plaque removal of 2.5 to 3 mm, whereas a filament length of around 1.5 mm only achieved a subgingival reach of about 1.5–2 mm. On average, a subgingival plaque removal of about 2 to 2.5 mm was assumed when using IDBs of these two sizes (see ‘Situation from 1970 to 1980’).

Until 1990, various IDB systems were available, but their designs were not fully declared. It took well over 30 years for the first systematic reviews to appear. These early studies generally favoured IDBs as they had been shown to be superior to other interdental cleaning aids. Despite different study designs, there was a consensus that IDBs performed better at plaque removal than, for example, dental floss, so they were increasingly recommended.

Over the last 10 years, however, conclusions from systematic reviews have become more cautious. Although the importance of IDBs is still recognised, the limited evidence base for their efficacy is increasingly pointed out. Recent meta-analyses have presented and discussed novel cleaning aids such as RPs with rubber spikes or lamellae. However, the strength of the available studies is still too limited to make clear recommendations.

The 2006 ISO standard represented a significant step forward in IDB development as it introduced defined criteria for size, design, and testing for the first time. This made it possible to establish quality benchmarks and facilitated the comparison of insertion resistance based on PHD values. Nevertheless, certain aspects of the ISO standard remained ambiguous and potentially misleading. For example, the current ISO standard still categorises IDBs into nine sizes with different PHD spectra without sufficient scientific justification. There are no studies in the literature that determine the specific spectrum of interdental spaces that an individual IDB can adequately clean based on its size. The inconsistent interval classifications give the impression of uniformity, which, however, is clearly distorted when evaluated on the basis of PHD values.

Furthermore, the rationale for the overall range of interdental space sizes observed in patients remains unclear. The measurement scale recommended by the ISO assumes a PHD value range of 0.6 to 3.5 mm, while a study from 2020 determined a wider range of 0.6 to 5.2 mm for commercially available IDBs^67^ (Fig 17). This indicates that the ISO scale needs to be updated. Only 33% of manufacturers adhere to ISO labelling standards for sizes and only 25% for PHD values.^67^ The ISO standard does not consider the length of the side bristles (filaments), which is crucial for understanding the potential range of an IDB. It also makes sense to update the data on the longitudinal and cross-sections of IDBs.

An important aspect is still the classification of the exact place of IDBs in terms of their purpose and effectiveness in oral hygiene. The question arises as to whether their primary function is to combat caries and gingivitis by removing supragingival plaque, or whether their design should also allow them to penetrate deeper into subgingival tooth areas. In cases of periodontitis characterised by bone loss and pocket formation, subgingival cleaning has traditionally been performed exclusively by dentists and dental hygienists, while little attention has been paid to sub-gingival home care by patients. This practice is based, among other things, on a 1992 study by Dahlén et al^17^ which found that the removal of supragingival plaque during oral hygiene at home has a positive effect on the subgingival microflora. However, the effect is limited.

Conventional toothbrushes are primarily designed for supragingival plaque removal and only have a limited subgingival reach, typically ≤1 mm.^16^ Dental floss and small interdental brushes (IDBs) can reach slightly deeper areas. The ideas about the reach of IDBs (approx. 2–2.5 mm below the gingival margin) originate mainly from the 1976 study by Waerhaug.^82^ Since only interdental brushes with a maximum filament length of 3 mm were used in this study (a fact that is regularly overlooked in the literature), a reassessment is required. Given the availability of IDBs with longer side bristles up to 7 mm, the question arises as to the potential benefits of more comprehensive subgingival cleaning by patients.

In summary, it can be said that the origins of IDBs remain obscure and their history has received little attention to date. In view of their potentially high significance, the study situation is still limited. The following conclusions can therefore only be described in part as uncertain and provisional:

The exact tooth areas to be cleaned with IDBs (supragingival, subgingival) have not yet been clearly defined. When selecting IDBs, an optimised combination of the PHD values and the lengths of sufficiently thick filaments appears to be decisive. The previous use of ISO sizes for selection purposes, on the other hand, may not be ideal as they can confuse and mislead the user. Newer IDB designs, especially those with a modified cross-section, could expand the range of indications, possibly also for subgingival cleaning. Home-use of IDBs can be part of Frugal Dentistry, which promotes dental methods and products that focus on core functions, take into account dental standards including risk-benefit assessment, consider the needs and expectations of the target audience and, last but not least, keep cost reduction in mind.^73^

The importance of IDBs can be characterised in the following five points:

In supragingival tooth cleaning of small interdental spaces (PHD values of up to approx. 1.0 mm) and shallow probing depths, the choice of tools used – dental flosses, IDBs or IRPs – may not be highly relevant with regard to the achievable clinical results due to the limited space available. Therefore, the patient’s preferences can primarily be considered.
At PHD values of 1.1 mm and above, IDBs with longer side bristles become more important, especially if cleaning is to extend to subgingival areas due to increased probing depths. The effectiveness of dental floss or IRPs decreases in these areas.
The aim should be to create IDB ranges that offer a broad spectrum of PHD values while minimizing the number of IDBs. The IDBs should increase continuously and evenly in defined PHD intervals, whereby it still needs to be scientifically clarified how large these intervals may be (eg, steps of two, three or even larger). The IS0 sizes are not appropriate in their current definition and should therefore be reconsidered. A scientifically validated range is therefore still pending. On such a basis, appropriate training of dental staff and patients would be possible.
For special requirements, designs should be developed that allow better access to niches (eg, to the subgingival spaces) that IDBs have so far been partially denied.
A development based on these proposals has the prospect of better exploiting the undeniably high potential of IDBs in a simple and cost-effective way. This could also make an important contribution to Frugal Dentistry.

## Acknowledgements

We are greatly indebted to Mr Andreas Haesler (Dental Museum, Schadraß, Germany), who not only provided exhibits for photographs but also conducted indispensable research, the results of which are shown in Figures 2 and 4–10. We would also like to extend our heartfelt thanks to Mr Benno Dörflinger (Brush Museum Todtnau, Germany) for the permission to reproduce Figure 3 and for the demonstration of the production of twisted brushes (Fig 11). The apparatus shown in Figure 12 is located in the German Brush Museum (Bechhofen a. d. Heide, Germany). For valuable information on the development of IDBs, we are also sincerely grateful to Mr Curt Bättig (topcaredent, Schönau, Germany) and Mr Ulrich Zahoransky (Zahoransky, Todtnau, Germany).
